# One Label or Two? Linguistic Influences on the Similarity Judgment of Objects between English and Japanese Speakers

**DOI:** 10.3389/fpsyg.2017.01637

**Published:** 2017-09-26

**Authors:** Takahiko Masuda, Keiko Ishii, Koji Miwa, Marghalara Rashid, Hajin Lee, Rania Mahdi

**Affiliations:** ^1^Department of Psychology, University of Alberta, Edmonton, AB, Canada; ^2^Department of Psychology, Kobe University, Kobe, Japan; ^3^Department of Linguistics, Eberhard Karls University of Tübingen, Tübingen, Germany; ^4^Department of Psychiatry, University of Alberta, Edmonton, AB, Canada

**Keywords:** similarity judgment, ordinary objects, Japanese speakers, English speakers, Whorfian hypothesis

## Abstract

Recent findings have re-examined the linguistic influence on cognition and perception, while identifying evidence that supports the Whorfian hypothesis. We examine how English and Japanese speakers perceive similarity of pairs of objects, by using two sets of stimuli: one in which two distinct linguistic categories apply to respective object images in English, but only one linguistic category applies in Japanese; and another in which two distinct linguistic categories apply to respective object images in Japanese, but only one applies in English. We conducted four studies and tested different groups of participants in each of them. In Study 1, we asked participants to name the two objects before engaging in the similarity judgment task. Here, we expected a strong linguistic effect. In Study 2, we asked participants to engage in the same task without naming, where we assumed that the condition is close enough to our daily visual information processing where language is not necessarily prompted. We further explored whether the language still influences the similarity perception by asking participants to engage in the same task basing on the visual similarity (Study 3) and the functional similarity (Study 4). The results overall indicated that English and Japanese speakers perceived the two objects to be more similar when they were in the same linguistic categories than when they were in different linguistic categories in their respective languages. Implications for research testing the Whorfian hypothesis and the requirement for methodological development beyond behavioral measures are discussed.

## Introduction

For many decades, researchers have examined whether linguistic categories determine, constrain, or influence human thoughts (e.g., Hunt and Agnoli, 1991; Lucy, 1992; Gumperz and Levinson, 1996; Bowerman and Levinson, 2001; Boroditsky, 2003; Gentner and Goldin-Meadow, 2003; Chiu et al., 2007; Malt and Wolff, 2010). This question has been discussed under the rubric of *linguistic relativity* or the *Whorfian hypothesis* (Sapir, 1921; Whorf, 1956). However, Whorf’s original concept of linguistic determinism has been criticized for lack of strong empirical evidence (Pinker, 1994, 1997, 2007). Scholars also disagree on a weaker version of the hypothesis, which asserts that language influences psychological processes such as cognition and perception, and no decisive conclusion has been reached (e.g., Boroditsky, 2001; January and Kako, 2007).

On one hand, the findings of many empirical studies in cognitive sciences suggest that the influence of linguistic categories on cognition and perception is minimal, favoring psychological universals (Berlin and Kay, 1969; Kay and McDaniel, 1978; Regier et al., 2005; Chen, 2007). On the other hand, recent studies have re-examined these findings, and have provided evidence in support of the hypothesis in the domains of categorization (Ji et al., 2004; Boutonnet et al., 2012), color perception (e.g., Davidoff et al., 1999; Roberson et al., 2000; Winawer et al., 2007; Roberson et al., 2008; Thierry et al., 2009; Roberson, 2012; Hu et al., 2014), time perception (e.g., Scott, 1989; Boroditsky, 2001; Casasanto and Boroditsky, 2008; Boroditsky et al., 2011; Fuhrman et al., 2011; Lai and Boroditsky, 2013), spatial perception (e.g., Levinson, 1996, 2003; Li and Gleitman, 2002; Majid et al., 2004; Choi and Hattrup, 2012), shape and substance (e.g., Lucy and Gaskins, 2001; Saalbach and Imai, 2012), grammatical effect of gender (e.g., Boroditsky et al., 2003; Boutonnet et al., 2012; Imai et al., 2014), and sound pitch perception (e.g., Dolscheid et al., 2013). In line with these latter studies, the current paper examines the effect of linguistic categories on the similarity judgment of ordinary objects.

Even early discussions among scholars referred to the effect of language on the perception of ordinary objects (e.g., Whorf, 1956; Jackson, 1991). However, to date, few researchers have tried to assess exactly how people in different language communities perceive similarities among ordinary concepts. This insufficiency of empirical research might be attributable to researchers being content with anecdotally reporting one or two examples of categorical differences in ordinary objects (e.g., the vocabularies related to the concept “snow”), while presupposing that identifying such differences provides evidence in support of the Whorfian hypothesis—rather than empirically testing the effect by using a sufficient number of stimuli. To overcome this deficiency, it is necessary to test the effect in a more systematic and comprehensive manner.

**FIGURE 1:**
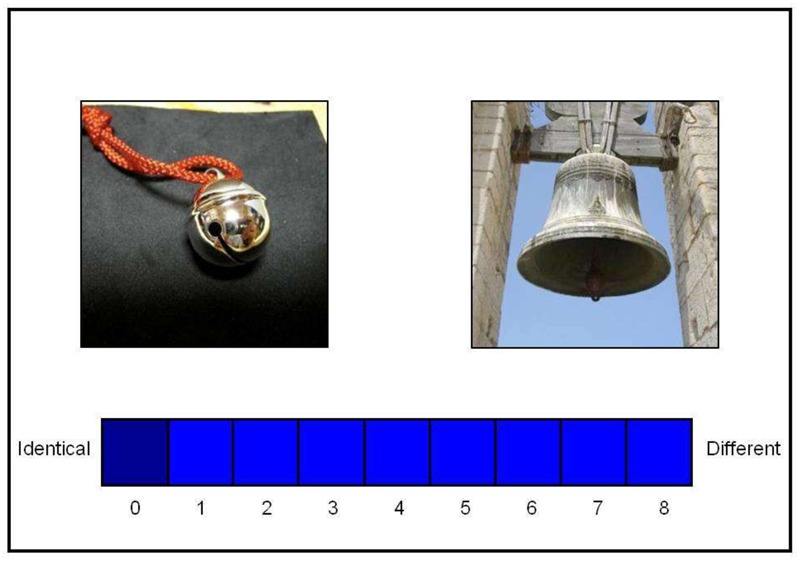
An example of pairs of images, and a scale.

We assume that people conceptualize objects along the lines drawn between existing categories in their native language. That is, if two concepts fall into the same linguistic category, the perception of similarity between these objects would be stronger than if the two concepts fall into different linguistic categories. For example, in Japanese, the kind of bell found in a bell tower generally corresponds to the word *kane*—a large bell—which is categorically different from a small bell, *suzu* (**Figure 1**). However, in English, these two objects are considered to belong within the same linguistic category, “bell.” Therefore, we might expect English speakers to perceive these two objects as being more similar than would Japanese speakers. Similarly, in English, a *bean* and a *pea* belong to two different linguistic categories. In contrast, a single linguistic category, “*mame*,” is applied to these two concepts in Japanese. It would therefore be reasonable to assume that Japanese speakers would perceive more similarity between these concepts than English speakers do. However, if our assumption is wrong, and the differences in linguistic categories do not affect people’s similarity perception of the two concepts, the perceptions of Japanese and English speakers would not differ from each other. We maintain that investigating such cross-language diversity in the relationship between concepts and corresponding linguistic categories will allow researchers to tangibly test Whorf’s speculation about the influence of language on people’s perception of reality (e.g., Whorf, 1956; Lucy, 1997; Boroditsky, 2003).

Toward this end, we devised a set of experimental stimuli consisting of pairs of images of ordinary objects, and we applied a common methodology in the field of cognitive sciences, cross-linguistically examining performance of cognitive tasks by speakers who use different native languages (Bowerman and Levinson, 2001; Gentner and Goldin-Meadow, 2003; Malt and Wolff, 2010). We conducted four studies, in which we asked Japanese and English speakers to engage in a similarity judgment task (Baayen and Boroditsky, 2004). In this task, participants were presented with an image depicting a pair of ordinary objects, and were asked to rate the level of similarity between these objects.

All of the procedures below followed the ethical standards of research with human behavioral data, and has been approved by the IRB committee at the authors’ institutions (#1927 Kan-08-11-022). In Study 1 (the naming task), we asked participants to engage in the task while naming concepts of respective objects before making a similarity judgment, and then we assessed whether the linguistic categories influenced their perception of similarity between the objects. We assumed that the task demand to name objects would make participants apply linguistic information to the task at hand, and therefore the linguistic effect would be clearly observable. In Study 2 (the non-naming, neutral task), we asked participants to engage in the same task without naming the concepts, in order to assess whether the influence of linguistic categories on the similarity judgment are observable even when participants do not explicitly state the linguistic categories of respective concepts. Although the experimental sessions were done in a self-paced manner and therefore did not impose any time control, we assumed that this condition is similar to what people access with visual information in everyday life, and therefore the most ecologically valid condition. We assume that, if the linguistic effect is observed in this condition, it would be supportive evidence for the linguistic relativity hypothesis. In Studies 3 and 4, we explore the linguistic effect on the similarity judgment while basing their decision on the visual similarity (Study 3) and functional similarity (Study 4) of pairs of ordinary objects. We assume that the visual similarity judgment would make participants engage in superficial observation of pictorial images, and therefore would require a relatively lower-level cognitive process, and the functional similarity judgment would make participants engage in accessing the meaning of the objects, which would require a higher-level cognitive process. If linguistic effects relevant to the present study are higher-level linguistic activation analogous to word naming, we assume that such task demands to perceive visual and functional similarity would attenuate the effect of linguistic categories on the similarity judgment of pairs of ordinary objects, albeit to different extents (i.e., more attenuation for Study 3 than Study 4).

We acknowledge that the current behavioral data could only test whether people access linguistic information even when they were not explicitly asked to do so, intending to associate this findings to cognitive psychology where researchers examine to what extent the top–down process influences our perception and cognition (Bruner, 1957, 1990; Cole and Scribner, 1974; Chun and Wolfe, 2001), and social psychology where researchers examine to what extent the priming words influence our social judgment (Oyserman and Lee, 2008). Therefore, the current studies provide only partial evidence against the criticism that, in order to show the effect of language, one must control people’s accessibility to language references (Pinker, 1994, 1997, 2007; Gleitman and Papafragou, 2005). However, we aimed to provide a behavioral, ecologically valid framework as a starting point for future neuroscientific research that focuses on participants’ automatic responses to stimuli while controlling for their accessibility to language references (e.g., Thierry, 2016 for review).

## Study 1

### Method

#### Participants

Thirty-one native English speakers (14 women, 17 men, *M*_age_ = 19.19, *SD* = 1.47) at the University of Alberta in Canada, and 29 native Japanese speakers (16 women, 13 men, *M*_age_ = 19.07, *SD* = 0.84) at Kobe University in Japan, took part in this study. Canadian participants received a course credit, and Japanese participants received $10 as an honorarium. All participants had normal or corrected-to-normal vision.

In order to measure the general fluency in English and Japanese among student bodies in both Japanese and Canadian data collection sites, we recruited Japanese and Canadian participants. These participants were different from those who engaged in the *perceptual* tasks reported later. Thirty-four Canadians and thirty Japanese participants subjectively judged their reading, listing, and writing ability in English and Japanese based on a 7-point Likert Scale (1 = Not at all, 7 = Native Level). We collapsed these three variables in each language into two variables: Average fluency in English and in Japanese. We also created a variable for participants’ fluency in a foreign language (i.e., Canadians’ fluency in Japanese, and Japanese’s fluency in English). As expected, the results indicated that Japanese thought that they were more fluent in Japanese than their Canadian counterparts (*M_JPN_* = 6.66, *SD_JPN_* = 0.52, *M_CND_* = 1.22, *SD_CND_* = 0.68), *t*(62) = 35.51, *p* < 0.001. Contrary, Canadians thought that they were more fluent in English than their Japanese counterparts (*M_CND_* = 6.97, *SD_CND_* = 0.17, *M_JPN_* = 3.64, *SD_JPN_* = 0.87), *t*(30.99) = 20.57, *p* < 0.001. Japanese thought that they were more fluent in Japanese (*M* = 6.66, *SD* = 0.52) than in English (*M* = 3.64, *SD* = 0.87), *t*(29) = 15.11, *p* < 0.001. Contrary, Canadians thought that they were more fluent in English (*M* = 6.97, *SD* = 0.17) than in Japanese (*M* = 1.22, *SD* = 0.68), *t*(33) = 48.43, *p* < 0.001. Finally, Japanese participants’ subjective fluency in English is better than Canadian participants’ subjective fluency in Japanese (*M_JPN_* = 3.64, *SD_JPN_* = 0.87, *M_CND_* = 1.22, *SD_CND_* = 0.68), *t*(54.74) = 12.31, *p* < 0.001, the results of which, we assumed, are attributable to the fact that the Japanese education system emphasizes English learning for the college entrance examination, while in Canadian school’s Japanese learning is optional.

#### Materials

Stimuli comprised pairs of pictorial objects grouped into two sets, according to how the objects were named in Japanese and English respectively. Two different pictures were prepared per each object to minimize superficial confound induced by different picture varieties (total 82 pairs). For each object, we selected images from object databases on the basis of the following criteria: high recognisability, high familiarity, weak cultural markedness, and low background complexity. Furthermore, from 41 pairs of stimuli prepared, we maximized the number of usable stimuli to minimize potential confound arising from particular semantic categories. In total, 18 pairs of objects were selected as the Distinct in Japanese (DJ) stimuli (represented by two distinct words in Japanese but not in English), and 16 pairs of words were selected as the Distinct in English (DE) stimuli (represented by two distinct words in English but not in Japanese) (see **Table 1**). Seven pairs of stimuli were eliminated either because both Japanese and English speakers equally dissected the concepts, or because most Japanese participants did not know the names of the objects. We also prepared six filler stimuli showing two identical pictures of ordinary objects. These items require the highest similarity score (i.e., identical) and thus facilitate all participants to use the scale in a similar, cross-culturally comparable manner (see Supplementary Data Sheet 1 for the list of stimuli).

**Table 1:** List of paired objects.

**Objects distinct in Japanese concepts, but not in English concepts (DJ)**
(1) *fukuro–kaban* (*plastic bag–bag*), (2) *gen–ito* (music string–string), (3) *geto–mon* (*gate–large gate*), (4) *gunte–gomutebukuro* (*gardening gloves–rubber gloves*), (5) *hake–fude* (*paint brush–writing brush*), (6) *hei–saku* (*large wood fence–metal fence*), (7) *jaguchi–totte* (*water faucet handle–handle on a cup*), (8) *kankisen–senpuki* (*exhaust fan–room fan*), (9) *kikyu–fusen* (*large balloon–small balloon*), (10) *kitte–hanko* (*postage stamp–stamp*), (11) *mizu–oyu* (*cold water–hot water*), (12) *naifu–hocho* (*knife–large knife*), (13) *shokkaku–antena* (*insect antenna–electronic antenna*), (14) *kara–kora* (*snail shell–turtle shell*), (15) *suiheisen–chiheisen* (*horizon over water–horizon over land*), (16) *suzu–kane* (*large bell–small bell*), (17) *tsubasa–hane* (*large wing–small wing*), (18) *ude–hijikake* (*arm–arm part of an armchair*).
**Objects distinct in English concepts, but not in Japanese concepts (DE)**
(1) *beak–bill* (*kuchibashi–kuchibashi*), (2) *beans–peas* (*mame–mame*), (3) *breadcrust–ear* (*mimi–mimi*), (4) *bubbles–foam* (*awa–awa*), (5) *chair–stool* (*isu–isu*), (6) *crab claw–scissors* (*hasami–hasami*), (7) *fang–tusk* (*kiba–kiba*), (8) *clock hand–needle* (*hari–hari*), (9) *horns–antlers* (*tsuno–tsuno*), (10) *mouse–rat* (*nezumi–nezumi*), (11) *mustache–beard* (*hige–hig*e), (12) *fingernail–claw* (*tsume–tsume*), (13) *thumb–big toe* (*oyayubi–oyayubi*), (14) *trunk–nose* (*hana–hana*), (15) *watch–clock* (*tokei–tokei*), (16) *web–nest* (*su–su*).

*( ) = corresponding concepts.*

For future research, interested readers are encouraged to take a look at the picture stimuli in Supplementary Data Sheet 1 and the corresponding words in Supplementary Table 1. The Supplementary Table 1 contains ZIPF word frequency scores, mora counts, syllable counts, and phonological edit distances. A phonological edit distance indicates how many operations are minimally required to transform one word form to another (Levenshtein, 1966), in terms of their phonological transcriptions. This was calculated in R (R Core Team, 2016) by means of the *sdists* function available in the R package *cba* (Buchta and Hahsler, 2009), with the weights of deletion, insertion, match, and replacement set to 1, 1, 0, 1, respectively. In transcribing Japanese words, the flap /tailr/ was used to encode English approximants /r/ and /l/. /Φ/ was used for a voiceless bilabial fricative, and vowels and consonants were repeated to encode the Japanese-specific moraic long vowels, moraic nasals, and moraic obstruents. In the case of a trial with a *bell* (Japanese *suzu*) and a *bell* (Japanese *kane*), for example, the phonological distance between the left and right English words is 0, while that of Japanese words is 4 (i.e., all four phonemes need to be replaced). Although phonological edit distance might have affected the results, particularly in Experiment 2, there was no significant difference between Japanese phonological edit distance in DJ condition and English phonological edit distance in DE condition, *t*(32) = 1.69, *p* = 0.10. This insignificant phonological distance remained unchanged when the difference was based on Japanese mora counts in the DJ condition and English syllable counts in the DE condition, *t*(32) = 1.34, *p* = 0.19.

In order to examine the word frequency level used for the study, we referred to the SUBTLEX corpus (Brysbaert and New, 2009) and BCCWJ corpus (Maekawa et al., 2014). Both corpuses allow us to examine the frequency of the target words appearing per million words. It was found that all of our Japanese stimuli (median = 9.86 per million, IQR = 15.9, range = 0.67:232.39) fall into the top 6% of all words in BCCWJ corpus and that the English stimuli (median = 20.87, IQR = 38.31, range = 0.56:516.18) fall into the top 31% in the SUBTLEX corpus, indicating that the stimuli sets prepared for the current study are fairly representative for words in their respective languages. Because raw frequency counts distribute with a long right tail, to study frequency effects on cognitive processes more precisely, we opted for the ZIPF transformation (Van Heuven et al., 2014). The mean ZIPF score for our Japanese stimuli was 3.97 (*SD* = 0.52, range = 2.82: 5.37) and that for our English stimuli was 4.33 (*SD* = 0.58, range = 2.75: 5.71), with the latter being significantly higher than the former, *t*(134) = 3.88, *p* < 0.001.

To check the equivalence of stimuli familiarity, 17 Canadians and 16 Japanese who were independent from the main participants judged the familiarity of each image based on a 7-point Likert Scale (1 = Not at all, 7 = very much). We collapsed their responses into three categories: DJ familiarity (represented by two distinct words in Japanese but not in English), DE familiarity (represented by two distinct words in English but not in Japanese), and Identical Image familiarity. The results indicated that, overall, Canadians were more likely than Japanese to think that images were familiar to them in DJ (*M_CND_* = 4,49, *SD_CND_* = 0.56, *M_JPN_* = 3.87, *SD_JPN_* = 0.54), *t*(31) = 3.24, *p* = 0.003, DE (*M_CND_* = 4.54, *SD_CND_* = 0.52, *M_JPN_* = 4.06, *SD_JPN_* = 0.58), *t*(31) = 2.50, *p* = 0.018, and Identical Images (*M_CND_* = 4.79, *SD_CND_* = 0.33, *M_JPN_* = 4.05, *SD_JPN_* = 0.64), *t*(31) = 4.22, *p* < 0.001. We maintain that the results are due to the fact that all images were selected from English-based free photo pages. Nonetheless, both Japanese’s and Canadians’ average scores reached above 4 out of 5 (*M_CND_* = 4.61, *SD_CND_* = 0.43, *M_JPN_* = 4.00, *SD_JPN_* = 0.55). Therefore, we concluded that these stimuli are usable for testing Japanese and Canadians’ language effect on perception.

To check the equivalence of concreteness (how much participants visualize the image concretely from target words), 17 Canadians and 14 Japanese who were independent from the main participants judged the concreteness of each words based on a 7-point Likert Scale (1 = Not at all, 7 = very much). In the DJ stimuli, Japanese judged two words, and the average scores of these two words were compared to a single English word judged by Canadians. In the DE stimuli, Canadians judged two words, and the average scores of these two words were compared to a single Japanese word judged by Japanese. We then collapsed their responses into three categories: DJ concreteness, DE concreteness, and Identical Image concreteness. The results indicated that there were no linguistic variations in concreteness judgment between Canadians and Japanese in DJ (*M_CND_* = 4,64, *SD_CND_* = 0.47, *M_JPN_* = 4.52, *SD_JPN_* = 0.36), *t*(29) = 0.76, *p* = 0.452, DE (*M_CND_* = 4.60, *SD_CND_* = 0.43, *M_JPN_* = 4.70, *SD_JPN_* = 0.30), *t*(29) = 0.71, *p* = 0.483, and Identical Images (*M_CND_* = 4.71, *SD_CND_* = 0.33, *M_JPN_* = 4.73, *SD_JPN_* = 0.64), *t*(31) = 0.16, *p* = 0.878. Therefore, we concluded concreteness of the target words were equivalent for Japanese and Canadians.

Japanese data were collected in Japan, and English data in Canada; we created similar experimental settings in both places. All the pairs of stimuli were randomly presented on a 17-inch computer screen (1024 × 768 pixels) using PsyScope on a Macintosh computer (see **Figure 1**).

#### Procedure

Each participant was individually escorted to an experimental cubicle. Participants were instructed that the task was to write down the names of the two objects on a sheet, and then rate how similar/different the objects were by clicking the appropriate point on a 9-point scale ranging from 0 (*identical*) to 8 (*extremely different*). Participants engaged in 12 practice trials before the experimental trials. In each trial, the participants saw a sequence of countdowns and an asterisk (3, 2, 1, ^∗^) followed immediately by a pair of pictures. The entire experimental session took 40 min on average (20 min on average for actual trials). Upon completion of the experiment, the participants were asked to fill out a questionnaire about their demographic information. Finally, participants were thanked for their participation and debriefed.

In each trial, Japanese and English speakers were presented with a pair of images. There were two types of pairing. In the DJ pairs, two concepts fell into one linguistic category in English but two linguistic categories in Japanese (e.g., *bell–bell* in English vs. *kane–suzu* in Japanese). In the DE pairs, two concepts were in one category in Japanese but in two categories in English (e.g., *mame–mame* in Japanese vs. *beans–peas* in English). In Study 1 (the naming condition), native Japanese and English speakers judged the similarity of the images, but first they explicitly named the objects, which served to verify the influence of language on judgment.

### Results and Discussion

**FIGURE 2:**
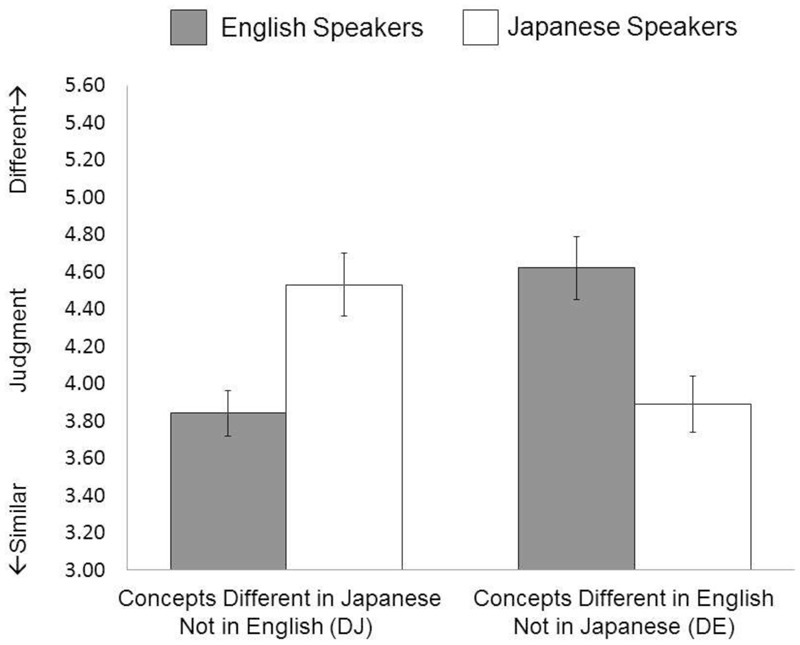
Results of Study 1.

Unexpected naming divided the total number of naming data, indicating that 8.15% of Japanese participants’ judgments and 8.24% of English participants’ judgments were labeled differently from our expected categorization. Therefore, we analyzed the data by excluding these unexpected accounts. A 2 (Language Group: English speakers vs. Japanese speakers) × 2 (Linguistic Category: DE vs. DJ) analysis of variance (ANOVA) on subjective similarity judgment identified a significant interaction between language group and linguistic category, *F*(1,58) = 86.43, *p* < 0.001, $\eta_{p}^{2}$ = 0.598. Simple effect analyses revealed that English speakers perceived the two objects to be more similar when they were in the same linguistic categories (*M* = 3.84, *SD* = 0.65) than when they were in the different linguistic categories (*M* = 4.64, *SD* = 0.94) in English, *t*(30) = 6.36, *p* < 0.001. Similarly, Japanese speakers perceived the two objects to be more similar when they were in the same linguistic categories (*M* = 3.86, *SD* = 0.78) than when they were in different linguistic categories (*M* = 4.62, *SD* = 0.91) in Japanese, *t*(1,28) = 6.94, *p* < 0.001. The similarity perception of both DJ and DE stimuli differed across languages: English speakers were more likely than their Japanese counterparts to perceive that two DJ objects were similar, *t*(58) = 3.83, *p* < 0.001, whereas Japanese speakers were more likely than English speakers to perceive that two DE objects were similar, *F*(1,58) = 9.94, *p* < 0.001 (see **Figure 2**).

As expected, the results indicated that the linguistic categories indeed influenced participants’ similarity judgments. Both Japanese speakers and English speakers judged the pairs of objects to be more similar when the objects were in the same linguistic category than when they were in different linguistic categories. This evidence supports the notion that linguistic category divergently influences people’s similarity perception of two objects across different languages, producing a linguistically different reality even though speakers are presented with the same images of objects. These results are, however, not surprising, because linguistic information was explicitly activated. The question relevant to Whorfian hypothesis is whether linguistic information contributes without presence of explicit linguistic activation.

## Study 2

In Study 2, we investigated whether the findings we reported in Study 1 were replicable when Japanese and English speakers engaged in the same task while not naming the objects. We reasoned that, if speakers’ similarity perception was still influenced by the linguistic categories even when they were not explicitly accessed, it would be evident that the effect of linguistic categories is robust. But, if the effect was not present, the results observed in Study 1 would be attributable to the participants’ explicit engagement in accessing linguistic categories of the target objects.

### Method

#### Participants

Twenty-seven native English speakers (13 women, 14 men, *M*_age_ = 19.48, *SD* = 2.44) were recruited at the University of Alberta in Canada, and 32 native Japanese speakers (18 women, 14 men, *M*_age_ = 18.87, *SD* = 0.70) were recruited at Kobe University in Japan. Canadian participants in Canada received a course credit, and Japanese participants received $10 as an honorarium. All participants had normal or corrected-to-normal vision.

#### Materials and Procedure

The same materials and procedure used in Study 1 were applied to Study 2, except that participants were asked to rate the similarity/difference without being asked to write down the names of the objects (the non-naming condition).

### Results and Discussion

**FIGURE 3:**
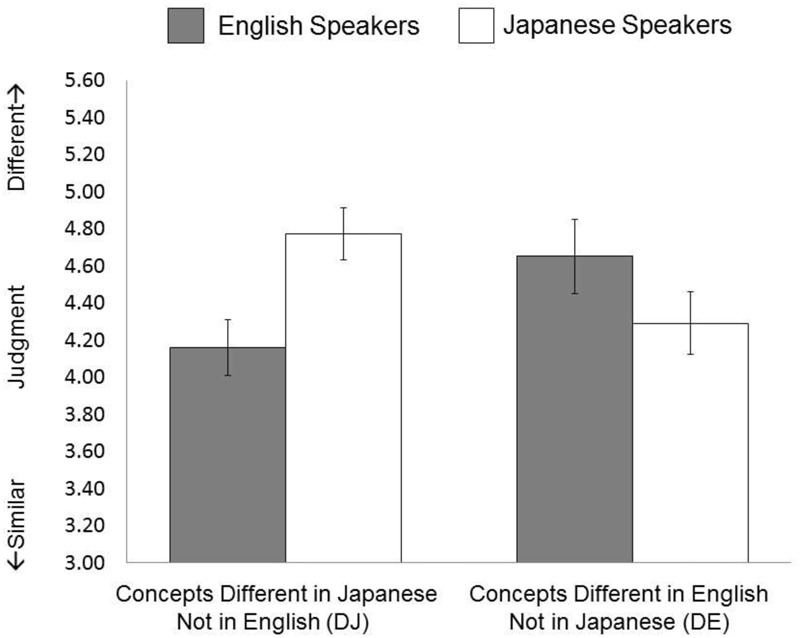
Results of Study 2.

A new group of participants engaged in the same task as in Study 1, but were not asked to name the objects; this allowed us to test whether the effect was observable even when the stimuli were not explicitly coded using language. A 2 (Language Group: English speakers vs. Japanese speakers) × 2 (Linguistic Category: DE vs. DJ) ANOVA on subjective similarity judgment identified a significant interaction between language group and linguistic category, *F*(1,57) = 42.69, *p* < 0.001, $\eta_{p}^{2}$ = 0.428. Simple effect analyses revealed that English speakers perceived the two objects to be more similar when they were in the same linguistic category (*M* = 4.16, *SD* = 0.79) than when they were in different linguistic categories (*M* = 4.65, *SD* = 1.03) in English, *t*(26) = 4.70, *p* < 0.001. Similarly, Japanese speakers perceived the two objects to be more similar when they were in the same linguistic categories (*M* = 4.29, *SD* = 0.96) than when they were in different linguistic categories (*M* = 4.77, *SD* = .81) in Japanese, *t*(31) = 4.59, *p* < 0.001. As for the differences in similarity perception of DJ and DE stimuli across languages, English speakers were more likely than their Japanese counterparts to perceive that two DJ objects were similar, *t*(57) = 2.61, *p* = 0.012. Japanese speakers tended to be more likely than English speakers to perceive that two DE objects were similar; however, the difference was not statistically significant, *t*(57) = 1.51, *p* = 0.146 (see **Figure 3**).

In order to test the differences in the magnitude of the language effect, we computed a new value by subtracting the participants’ judgment values of the DE (English has two labels) stimuli from these of the DJ (Japanese has two labels) stimuli, and merged the dataset of Study 1 and 2. Then we conducted a 2 (Language: Japanese vs. English) × 2 (Condition: Study 1 vs. Study 2) ANOVA, applying to the new variable. Results indicated that there is an interaction between language and condition, *F*(1,57) = 7.04, *p* = 0.009, $\eta_{p}^{2}$ = 0.058, suggesting that the magnitude of the language effects was slightly attenuated in Study 2 (*M_jpn_* = 0.47, *SD_jpn_* = 0.59 vs. *M_eng_* = -0.49, *SD_eng_* = 0.54) compared Study 1 (*M_jpn_* = 0.77, *SD_jpn_* = 0.59 vs. *M_eng_* = -0.80, *SD_eng_* = 0.70).

The results overall replicated those of Study 1. These findings suggest that, without direct naming of the target objects, both Japanese and English speakers are still influenced by the linguistic categories of their native language when they judge similarity of pairs of ordinary objects.

## Study 3

To further examine whether the effect of linguistic categories was attenuated or still sustained when participants’ attention was directed to lower-level visual features of the pairs of objects, we asked participants to engage in the similarity judgment task by asking them to focus on the visual similarity of the pairs of objects.

### Method

#### Participants

Twenty-six native English speakers (10 women, 16 men, *M*_age_ = 18.85, *SD* = 1.46) were recruited at the University of Alberta in Canada, and 33 native Japanese speakers (20 women, 13 men, *M*_age_ = 18.76, *SD* = 1.52) were recruited at Kobe University in Japan.

#### Materials and Procedure

The same materials and procedure as in Study 1 were used, except that participants were asked to rate the similarity/difference basing their decisions on the visual similarity of the objects.

### Results and Discussion

**FIGURE 4:**
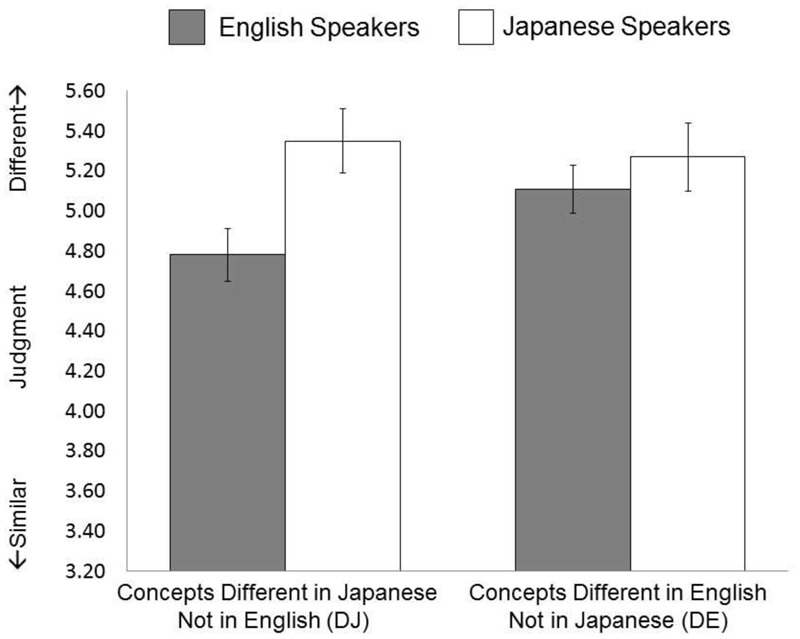
Results of Study 3.

A new group of native Japanese and English speakers engaged in the same task, but were asked to base their decision on the visual similarity of the objects. A 2 (Language Group: English speakers vs. Japanese speakers) × 2 (Linguistic Category: DE vs. DJ) ANOVA on subjective similarity judgment identified a significant interaction between language group and linguistic category, *F*(1,57) = 8.75, *p* = 0.004, $\eta_{p}^{2}$ = 0.133. Simple effect analyses revealed that English speakers perceived the two objects to be more similar when they were in the same linguistic categories (*M* = 4.78, *SD* = 0.71) than when they were in different linguistic categories (*M* = 5.12, *SD* = 0.59), *t*(25) = 3.57, *p* = 0.001. However, Japanese speakers’ similarity judgment values were about the same when the objects were in the same linguistic category (*M* = 5.27, *SD* = 1.00) as when they were in different linguistic categories (*M* = 5.35, *SD* = 0.93), *t*(32) = 0.79., *p* = 0.435. In addition, the similarity perception of DJ and DE stimuli differed across languages. That is, English speakers were more likely than Japanese speakers to perceive that two DJ objects were similar, *t*(57) = 2.54, *p* = 0.014, but there was no significant difference in the similarity judgment for DE stimuli between Japanese and English speakers, *t*(57) = 0.73., *p* = 0.468 (see **Figure 4**).

In order to test the differences in the magnitude of the language effect, we computed a new value by subtracting the participants’ judgment values of the DE (English has two labels) stimuli from these of the DJ (Japanese has two labels) stimuli, and merged the dataset of Studies 1 and 3. Then we conducted a 2 (Language: Japanese vs. English) × 2 (Condition: Study 1 vs. Study 3) ANOVA, applying to the new variable. Results indicated that there is an interaction between language and condition, *F*(1,118) = 28.84, *p* < 0.001, $\eta_{p}^{2}$ = 0.201, suggesting that the magnitude of the language effects was attenuated in Study 3 (*M_jpn_* = 0.07, *SD_jpn_* = 0.56 vs. *M_eng_* = -0.33, *SD_eng_* = 0.46) compared Study 1 (*M_jpn_* = 0.77, *SD_jpn_* = 0.59 vs. *M_eng_* = -0.80, *SD_eng_* = 0.70). We also conducted we conducted a 2 (Language: Japanese vs. English) × 2 (Condition: Study 2 vs. Study 3) ANOVA. Results indicated that there is an interaction between language and condition, *F*(1,117) = 7.98, *p* = 0.006, $\eta_{p}^{2}$ = 0.065, suggesting that the magnitude of the language effects was slightly attenuated in Study 3 (*M_jpn_* = 0.07, *SD_jpn_* = 0.56 vs. *M_eng_* = -0.33, *SD_eng_* = 0.46) compared Study 2 (*M_jpn_* = 0.47, *SD_jpn_* = 0.59 vs. *M_eng_* = -0.49, *SD_eng_* = 0.54).

Why were Japanese speakers more likely than English speakers to easily ignore the linguistic labels of the stimuli? One possibility is that Japanese speakers were more context-sensitive than English speakers (Masuda and Nisbett, 2001, 2006; Masuda et al., 2012, 2016; Senzaki et al., 2014, 2016). In fact, previous findings suggest that Japanese speakers are good at spontaneously allocating their attention to the intonation of words spoken aloud rather than the meaning of the words (Ishii et al., 2003). If so, it is possible that Japanese speakers are better able to direct their attention to the visual similarity, which results in effacing the language effects, whereas English speakers’ language effect still lingers in the visual condition. In fact, results of the main effect of culture in Study 3 indicated that, compared to English speakers, Japanese speakers tended to differentiate the pairs of objects. This pattern was marginally significant, *F*(1,57) = 2.96, *p* = 0.091, $\eta_{p}^{2}$ = 0.025, suggesting that Japanese speakers may apply the visual-based strategy to the similarity judgment better than English speakers.

## Study 4

To further examine whether the effect of linguistic categories was attenuated or still sustained when participants’ attention was directed to higher-level visual features of the pairs of objects, we asked participants to engage in the similarity judgment task by asking them to focus on the functional similarity of the pairs of objects.

### Method

#### Participants

Twenty-six native English speakers (12 women, 14 men, *M*_age_ = 18.73, *SD* = 1.40) were recruited at the University of Alberta in Canada, and 34 native Japanese speakers (24 women, 10 men, *M*_age_ = 18.65, *SD* = 0.73) were recruited at Kobe University in Japan.

#### Materials and Procedure

The same materials and procedure as in Study 1 were used, except that participants were asked to rate the similarity/difference while being asked to think of the functional similarity of the objects.

### Results and Discussion

**FIGURE 5:**
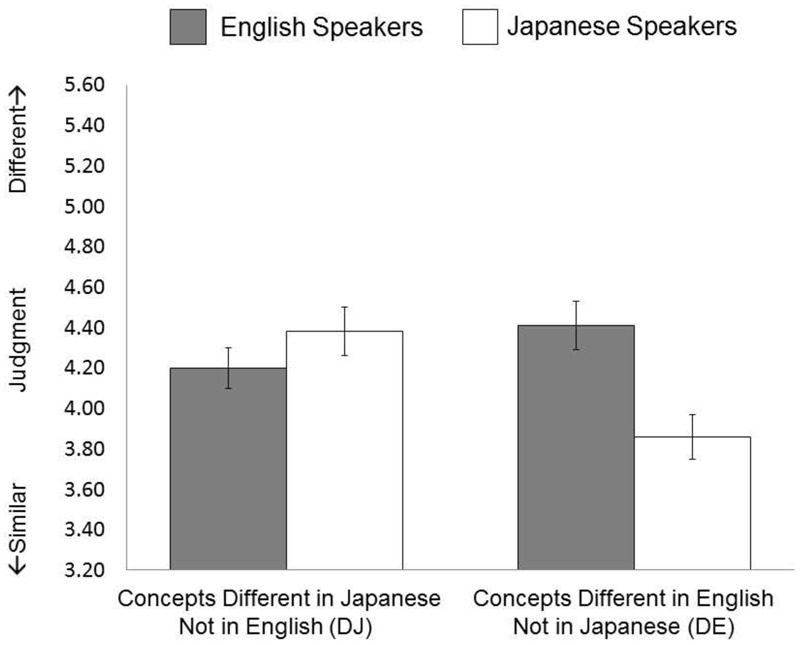
Results of Study 4.

A new group of native Japanese and English speakers engaged in the same task, but were asked to base their decision on the functional similarity of the objects. A 2 (Language Group: English speakers vs. Japanese speakers) × 2 (Linguistic Category: DE vs. DJ) ANOVA on subjective similarity judgment identified a significant interaction between language group and linguistic category, *F*(1,58) = 25.53, *p* < 0.001, $\eta_{p}^{2}$ = 0.306. Simple effect analyses revealed that when shown the same pairs, English speakers tended to perceive the two objects to be more similar when they were in the same linguistic categories (*M* = 4.20, *SD* = 0.55) than when they were in different linguistic categories (*M* = 4.41, *SD* = 0.64), but the difference was statistically marginal, *t*(25) = 1.97, *p* = 0.060. However, Japanese speakers perceived the two objects to be more similar when they were in the same linguistic categories (*M* = 3.86, *SD* = 0.63) than when they were in different linguistic categories (*M* = 4.38, *SD* = 0.72), *t*(33) = 5.40, *p* < 0.001. In addition, there was no significant difference in the similarity judgment for DJ stimuli between Japanese and English speakers, *t*(58) = 1.07, *p* = 0.289. However, Japanese speakers were more likely than their English counterparts to perceive that two DE objects were similar, *t*(58) = 3.27, *p* = 0.002 (see **Figure 5**).

Again, while the patterns of the similarity judgments were overall similar to those found in Study 1, the effect of linguistic category on similarity judgment was slightly attenuated when Japanese and English speakers paid attention to the functional similarity of the pairs of objects. Notably, English speakers tended to think that the pairs of objects in DE stimuli were functionally not so similar to each other, weakening the effect of the linguistic category. However, if we consider this result from another point of view, the English speakers’ response patterns observed in Study 1 indeed represented the fact that the linguistic category constrained English speakers to perceive similarity between objects beyond the perceived functional differences between them.

In order to test the differences in the magnitude of the language effect, we computed a new value by subtracting the participants’ judgment values of the DE (English has two labels) stimuli from these of the DJ (Japanese has two labels) stimuli, and merged the dataset of Studies 1 and 4. Then we conducted a 2 (Language: Japanese vs. English) × 2 (Condition: Study 1 vs. Study 4) ANOVA, applying to the new variable. Results indicated that there is an interaction between language and condition, *F*(1,119) = 14.01, *p* < 0.001, $\eta_{p}^{2}$ = 0.108, suggesting that the magnitude of the language effects was slightly attenuated in Study 4 (*M_jpn_* = 0.54, *SD_jpn_* = 0.57 vs. *M_eng_* = -0.21, *SD_eng_* = 0.55) compared Study 1 (*M_jpn_* = 0.77, *SD_jpn_* = 0.59 vs. *M_eng_* = -0.80, *SD_eng_* = 0.70). We also conducted a 2 (Language: Japanese vs. English) × 2 (Condition: Study 2 vs. Study 4) ANOVA. Results indicated that the interaction value did not reach statistical significance, *F*(1,119) = 1.30, *p* = 0.257, $\eta_{p}^{2}$ = 0.011, suggesting that the magnitude of the language effects was similar between Study 2 (*M_jpn_* = 0.47, *SD_jpn_* = 0.59 vs. *M_eng_* = -0.49, *SD_eng_* = 0.54) and Study 4 (*M_jpn_* = 0.54, *SD_jpn_* = 0.57 vs. *M_eng_* = -0.21, *SD_eng_* = 0.55).

Were Japanese speakers more likely than English speakers to easily ignore the linguistic labels of the stimuli? If the Japanese speakers were attentive to the context of the objects, they might differentiate the pairs of objects more than English speakers. However, at this time, there is no main effect of culture, *F*(1,58) = 1.49, *p* = 0.227, $\eta_{p}^{2}$ = 0.025. This means that this possibility may be weak for participants’ functional judgment. Nevertheless, it is advisable for future research to incorporate the effect of context sensitivity into the design in order to comprehensively elucidate the relationships between culture, language, and cognition (Imai and Masuda, 2013).

## General Discussion

The Whorfian hypothesis has been criticized by universalists who have argued for the independence of cognitive and perceptual processes from top–down processes, including language (Pinker, 1994, 1997, 2007; Pylyshyn, 1999; Fodor, 2008). However, previous behavioral data have provided evidence in favor of language effects on perception and cognition (e.g., Davidoff et al., 1999; Gilbert et al., 2006, 2008) and consistent neurophysiological evidence is now accumulating (Thierry et al., 2009; Mo et al., 2011). In the same vein, the current paper focuses on people’s perception of ordinary objects in relation to native language terminology and provides further behavioral evidence that people access linguistic categories even when they are not explicitly instructed to do so, which in turn influences their judgment.

Four studies (Two main studies and two exploratory studies) examined Japanese and English participants’ similarity perception of ordinary objects. The results suggest that explicit use of language during the task indeed influenced participants’ similarity judgments of target pairs of ordinary objects (Study 1). Furthermore, although the magnitude was attenuated, we observed the language effect on their similarity judgment even when participants were not explicitly requested to name the target pairs of ordinary objects (Study 2), when participants attended to the visual appearance of the target pairs of ordinary objects (Study 3), and when participants attended to the functions of the target pairs of ordinary objects (Study 4).

### Implications

The current paper has three major implications. First, differences in linguistic categorization of ordinary concepts are ubiquitous, and could be a major source of miscommunication between people who speak different languages. Shedding light on a long-lasting question in linguistics that has been dropped from the discourse, the current research on ordinary concepts revitalizes Whorf’s original concepts in a broader context. Second, the findings facilitate discussions about universality and specificity of language-related cognition and perception. Given the fact that both universality and language influence cognition, it is reasonable to assume that human cognition has both universal and language-specific aspects.

However, the Whorfian hypothesis has been debated largely in a black-and-white manner, with researchers supporting their extreme stances (e.g., universalism vs. relativism) with limited amounts of scientific evidence. Recently, an alternative theoretical framework has advocated a balanced view (e.g., Imai and Masuda, 2013; Imai et al., 2016), whereby researchers scrutinize the magnitude of the effect of linguistic categories on a variety of cognitive processes with a sufficient amount of empirical evidence (e.g., Saalbach and Imai, 2007; Regier and Kay, 2009; Imai and Saalbach, 2010), rather than overgeneralizing about or rejecting such an effect according to their ideological stance. In line with these investigations, the current paper addresses the issue of when, and to what extent, the effect of language categories on the similarity perception of ordinary objects is strengthened or attenuated, by assessing four different conditions.

Third, recent neuroscientific evidence has shown that linguistic effects on perception can be found even when linguistic reference is minimized, filtered, or even blocked (Thierry, 2016). For example, Boutonnet et al. (2012) recruited English speakers who make a distinction between cup and mug, and Spanish speakers who call both objects *taza*, and asked them to engage in a monitoring task in which where they were asked to report a target object (bowl). During the task, pictures of a cup or a mug were presented at a specific probability rates (e.g., 80% cup and 15% mug). Compared to Spanish speakers, English speakers who differentiate mug and cup showed enhanced differential visual mismatch negativity (vMMN) between the two objects, suggesting that they are more sensitive to the contrast between objects than their Spanish peers. The current study presents congruent behavioral findings which can serve as a basis for future neurophysiological investigations along the same lines.

### Limitations and Future Research

This paper has some limitations. First, although we attempted to create two comprehensive sets of stimuli, future studies should systematically cover a much wider range of stimuli in order to test the generalizability of the findings. Second, to advance this line of research, it is advisable to conduct similar tests focusing on speakers of languages other than English and Japanese. Third, the current paper did not include self-report scales that assess participants’ cognitive tendencies such as the self-construal scale (Singelis, 1994), the holism scale (Choi et al., 2007), and the dialectical self scale (Spencer-Rodgers et al., 2015; Unpublished). Future research should examine these variables, which may further elucidate cultural and individual variations in the similarity judgments. Fourth, in Studies 3 and 4, we instructed the task demand (the visual similarity in Study 3 and the functional similarity in Study 4) only once before the participants actually engaged in the task. Future research must investigate if the effect of language will be further attenuated when the task demand is reminded in each experimental trial. Fifth, in the current experiment we did not record participants’ metacognitive evaluation of their behavior during the testing session. Future research should include qualitative data analyses in order to better understand participants’ strategies to handle the task, and potential individual differences ensuing. Sixth, because we allowed participants to make similarity ratings at their own pace, we did not record reaction times. In order to assess participants’ reaction time, future research could resort to using a forced choice decision task, where participants are asked to judge whether images presented in pairs are similar or different, which would allow researchers to determine whether language effects are sustained under time constraints. Seventh, for the examination of linguistic relativity hypothesis, it is ideal to test fully monolingual individuals. However, the majority of Japanese participants learned English from the 7th grade, and English is a subject for the entrance examination for most Japanese universities. Similarly, the majority of Canadian participants are exposed to multilingual circumstances, and some of them may be exposed to Japanese. In order to know general students’ English and Japanese fluency in each data site, the current paper conducted two *post hoc* data collections. As expected, the results indicated that Japanese students scored their Japanese fluency higher than their English fluency, and Canadian students scored their English fluency higher than their Japanese fluency. Also, Japanese participants’ English fluency scores were higher than the Canadian participants’ scores of Japanese fluency, the results of which, we interpreted, is due to the Japanese education system. However, future research should directly collect the level of Japanese and English fluency from the actual participants who engaged in the perceptual tasks. Furthermore, future research should apply stricter criteria for participants’ level of foreign language fluency, especially when one assesses the effect of language on perception in bilingual speakers (e.g., Athanasopoulos et al., 2010).

Finally, and more importantly, although our purpose to provide evidence of the linguistic effect on the similarity judgment even participants are not explicitly told to use linguistic labels, it does not mean that the findings can completely answer the criticism against the linguistic relativity hypotheses (e.g., Pinker, 1994, 1997, 2007). Several researchers indeed maintain that language-specific differences in cognition and perception are mostly attributable to the fact that participants implicitly access the linguistic categories, and therefore, such findings cannot be used as true evidence in support of the Whorfian hypothesis (e.g., Gleitman and Papafragou, 2013); and that when the implicit linguistic labeling is inhibited, the effect of linguistic categories disappears (e.g., Roberson and Davidoff, 2000; Gilbert et al., 2006; Papafragou et al., 2008).

However, options for controlling linguistic activation during experimental tasks need to be examined in a more refined manner (Thierry, 2016). Recent neuroscientific evidence suggests that, perceptual and cognitive processes can be influenced by linguistic categories even when the accessibility to linguistic categories is limited, and thus that linguistic processes appear highly integrated with perceptual and cognitive processing (e.g., Thierry et al., 2009; Boutonnet et al., 2012; Lupyan, 2012; Imai et al., 2014). Although this issue is beyond the scope of the current paper, further research should devise methods to control automatic activation of linguistic categories, and test whether the effect of language on similarity judgment is still observable.

In summary, the current paper re-examined the Whorfian hypothesis—a long-lasting topic in anthropology, linguistics, psycholinguistics, and cognitive sciences—by demonstrating behavioral data that people’s similarity judgments of ordinary objects are indeed influenced by their own languages. We assumed that they spontaneously access linguistic references. This means that we have not yet provided convincing evidence that such an effect of language occurred without linguistic references. Nonetheless, we maintain that the findings contribute to further activate discussions and facilitate future neuroscientific research.

## Ethics Statement

This study was carried out in accordance with the recommendations of Human Research Ethics Board 2, University of Alberta with written informed consent from all subjects. All subjects gave written informed consent in accordance with the Declaration of Helsinki. The protocol was approved by Human Research Ethics Board 2, University of Alberta.

## Author Contributions

TM is the main author of the manuscript (70%). KM, MR, HL, RM, and KI contributed to conduct research, analyze data, and partially contributed to write this manuscript (10% respectively).

## Conflict of Interest Statement

The authors declare that the research was conducted in the absence of any commercial or financial relationships that could be construed as a potential conflict of interest.
