# Supplementary material for: One Label or Two? Linguistic Influences on the Similarity Judgment of Objects between English and Japanese Speakers
Source: Front Psychol. 2017 Sep 26;8:1637. doi: 10.3389/fpsyg.2017.01637 (PMC5623002; doi:10.3389/fpsyg.2017.01637)
Supplement: Supplementary file 1 [file Table_1.docx]

Table 1.

|  | Type | **English Label**  **[IPA]** | Freq | ZIPF | **Japanese Label 1 [IPA]** | Freq | ZIPF | **Japanese Label 2 [IPA]** | Freq | ZIPF |
| --- | --- | --- | --- | --- | --- | --- | --- | --- | --- | --- |
| 1 | DJ | **bag[bæg ]** | 24.84 | 5,088 | **fukuro[fukuɽo]** | 24.84 | 4,395 | **kaban[kabann]** | 11.98 | 4,078 |
| 2 | DJ | **string[strɪŋ]** | 4.94 | 4,320 | **gen[genn]** | 4.94 | 3,694 | **ito[ito]** | 25.42 | 4,405 |
| 3 | DJ | **gate[geɪt]** | 3.79 | 4,658 | **geto[geeto]** | 3.79 | 3,579 | **mon[monn]** | 26.3 | 4,420 |
| 4 | DJ | **gloves[glʌvz]** | 1.53 | 4,421 | **gunte[gunnte]** | 1.53 | 3,185 | **tebukuro[tebukuɽo]** | 7.1 | 3,851 |
| 5 | DJ | **brush[brʌʃ]** | 2.24 | 4,192 | **hake[hake]** | 2.24 | 3,350 | **fude[ɸude]** | 13.83 | 4,141 |
| 6 | DJ | **fence[fens]** | 8.24 | 4,265 | **hei[hei]** | 8.24 | 3,916 | **saku[saku]** | 7.65 | 3,884 |
| 7 | DJ | **handle[hændəl]** | 2.64 | 5,052 | **jaguchi[dʒagutʃi]** | 2.64 | 3,422 | **totte[totte]** | 3.48 | 3,542 |
| 8 | DJ | **fan[fæn]** | 1.81 | 4,691 | **kankisen[kannkisenn]** | 1.81 | 3,258 | **sempuki[sennpuuki]** | 3.11 | 3,493 |
| 9 | DJ | **balloon[bəlun]** | 0.67 | 4,126 | **kikyu[kikju]** | 0.67 | 2,826 | **fusen[ɸusenn]** | 4.21 | 3,624 |
| 10 | DJ | **stamp[stæmp ]** | 7.16 | 4,048 | **kitte[kitte]** | 7.16 | 3,855 | **hanko[hannko]** | 2.42 | 3,384 |
| 11 | DJ | **water[wɔtər ]** | 232.39 | 5,373 | **mizu[mizu]** | 232.39 | 5,366 | **oyu[oju]** | 26.8 | 4,428 |
| 12 | DJ | **knife[naɪf ]** | 15.69 | 4,672 | **naifu[naiɸu]** | 15.69 | 4,196 | **hocho[hootʃoo]** | 10.16 | 4,007 |
| 13 | DJ | **antenna[æntenə ]** | 1.58 | 3,450 | **shokkaku[ʃokkaku]** | 1.58 | 3,199 | **antena[anntena]** | 6.58 | 3,818 |
| 14 | DJ | **shell[ʃel]** | 8.92 | 4,274 | **snailkara[kaɽa]** | 8.92 | 3,950 | **kora[kooɽa]** | 1.54 | 3,188 |
| 15 | DJ | **horizon[həraɪzən ]** | 3.48 | 3,749 | **suiheisen[suiheisenn]** | 3.48 | 3,542 | **chiheisen[tʃiheisenn]** | 3.32 | 3,521 |
| 16 | DJ | **bell[bel]** | 5.43 | 4,720 | **suzu[suzu]** | 5.43 | 3,735 | **kane[kane]** | 9.72 | 3,988 |
| 17 | DJ | **wing[wɪŋ]** | 12.97 | 4,601 | **tsubasa[tsubasa]** | 12.97 | 4,113 | **hane[hane]** | 14.7 | 4,167 |
| 18 | DJ | **arm[ɑrm]** | 80.74 | 5,098 | **ude[ude]** | 80.74 | 4,907 | **hijikake[hidʒikake]** | 0.95 | 2,978 |

IPA = International Phonetic Alphabet, Freq = Frequency.

|  | Type | **Japanese Label [IPA]** | Freq | ZIPF | **English Label 1 [IPA]** | Freq | ZIPF | **English Label 2 [IPA]** | Freq | ZIPF |
| --- | --- | --- | --- | --- | --- | --- | --- | --- | --- | --- |
| 1 | DE | **kuchibashi[kutʃibaʃi]** | 3.74 | 3,573 | **beak[bik]** | 2.35 | 3,371 | **bill[bɪl ]** | 21.49 | 4,332 |
| 2 | DE | **mame[mame]** | 10 | 4 | **beans[binz]** | 21.27 | 4,328 | **peas[piz]** | 8.55 | 3,932 |
| 3 | DE | **mimi[mimi]** | 106.64 | 5,028 | **crust[krʌst]** | 3.74 | 3,573 | **ear[ɪər]** | 65.43 | 4,816 |
| 4 | DE | **awa[awa]** | 9.28 | 3,968 | **bubbles[bʌbəlz]** | 13.98 | 4,146 | **foam[foʊm ]** | 3.61 | 3,558 |
| 5 | DE | **isu[isu]** | 48.85 | 4,689 | **chair[ʧeər]** | 59.12 | 4,772 | **stool[stul ]** | 4.18 | 3,621 |
| 6 | DE | **hasami[hasami]** | 7.72 | 3,888 | **claw[klɔ]** | 8.8 | 3,944 | **scissors[sɪzərz]** | 7.04 | 3,848 |
| 7 | DE | **kiba[kiba]** | 4.54 | 3,657 | **fang[fæŋ]** | 1.78 | 3,250 | **tusk[tʌsk]** | 0.56 | 2,748 |
| 8 | DE | **hari[haɽi]** | 17.76 | 4,249 | **hand[hænd]** | 516.18 | 5,713 | **needle[nidəl]** | 17.08 | 4,232 |
| 9 | DE | **tsuno[tsuno]** | 1.09 | 3,037 | **horns[hɔrnz]** | 27.28 | 4,436 | **antlers[æntlərz]** | 1.19 | 3,076 |
| 10 | DE | **nezumi[nezumi]** | 14.4 | 4,158 | **mouse[maʊs]** | 19.18 | 4,283 | **rat[ræt]** | 49.24 | 4,692 |
| 11 | DE | **hige[hige]** | 14.1 | 4,149 | **mustache[mʌstæʃ]** | 5.29 | 3,723 | **beard[bɪərd]** | 13.37 | 4,126 |
| 12 | DE | **tsume[tsume]** | 21.68 | 4,336 | **nail[neɪl]** | 29.69 | 4,473 | **claw[klɔ]** | 8.8 | 3,944 |
| 13 | DE | **oyayubi[ojajubi]** | 12.38 | 4,093 | **thumb[θʌm]** | 16.49 | 4,217 | **toe[toʊ]** | 25.2 | 4,401 |
| 14 | DE | **hane[hana]** | 50.44 | 4,703 | **trunk[trʌŋk]** | 22.31 | 4,348 | **nose[noʊz]** | 74.51 | 4,872 |
| 15 | DE | **tokei[tokei]** | 20.69 | 4,316 | **watch[wɑʧ]** | 337.98 | 5,529 | **clock[klɑk]** | 61.47 | 4,789 |
| 16 | DE | **su[su]** | 19.68 | 4,294 | **web[web]** | 9.77 | 3,990 | **nest[nest]** | 11.55 | 4,063 |

IPA = International Phonetic Alphabet, Freq = Frequency.

|  | Type | **English Label**  **[IPA]** | Syllables | **Japanese Label 1 [IPA]** | Mora | **Japanese Label 2 [IPA]** | Mora | PED |
| --- | --- | --- | --- | --- | --- | --- | --- | --- |
| 1 | DJ | **bag[bæg ]** | 1 | **fukuro[fukuɽo]** | 3 | **kaban[kabann]** | 3 | 6 |
| 2 | DJ | **string[strɪŋ]** | 1 | **gen[genn]** | 2 | **ito[ito]** | 2 | 4 |
| 3 | DJ | **gate[geɪt]** | 1 | **geto[geeto]** | 3 | **mon[monn]** | 2 | 5 |
| 4 | DJ | **gloves[glʌvz]** | 1 | **gunte[gunnte]** | 3 | **tebukuro[tebukuɽo]** | 4 | 7 |
| 5 | DJ | **brush[brʌʃ]** | 1 | **hake[hake]** | 2 | **fude[ɸude]** | 2 | 3 |
| 6 | DJ | **fence[fens]** | 1 | **hei[hei]** | 2 | **saku[saku]** | 2 | 4 |
| 7 | DJ | **handle[hændəl]** | 2 | **jaguchi[dʒagutʃi]** | 3 | **totte[totte]** | 3 | 7 |
| 8 | DJ | **fan[fæn]** | 1 | **kankisen[kannkisenn]** | 5 | **sempuki[sennpuuki]** | 5 | 8 |
| 9 | DJ | **balloon[bəlun]** | 2 | **kikyu[kikju]** | 3 | **fusen[ɸusenn]** | 4 | 6 |
| 10 | DJ | **stamp[stæmp ]** | 1 | **kitte[kitte]** | 3 | **hanko[hannko]** | 3 | 6 |
| 11 | DJ | **water[wɔtər ]** | 2 | **mizu[mizu]** | 2 | **oyu[oju]** | 2 | 3 |
| 12 | DJ | **knife[naɪf ]** | 1 | **naifu[naiɸu]** | 3 | **hocho[hootʃoo]** | 4 | 7 |
| 13 | DJ | **antenna[æntenə ]** | 3 | **shokkaku[ʃokkaku]** | 4 | **antena[anntena]** | 4 | 7 |
| 14 | DJ | **shell[ʃel]** | 1 | **snailkara[kaɽa]** | 2 | **kora[kooɽa]** | 3 | 2 |
| 15 | DJ | **horizon[həraɪzən ]** | 3 | **suiheisen[suiheisenn]** | 6 | **chiheisen[tʃiheisenn]** | 5 | 2 |
| 16 | DJ | **bell[bel]** | 1 | **suzu[suzu]** | 2 | **kane[kane]** | 2 | 4 |
| 17 | DJ | **wing[wɪŋ]** | 1 | **tsubasa[tsubasa]** | 3 | **hane[hane]** | 2 | 6 |
| 18 | DJ | **arm[ɑrm]** | 1 | **ude[ude]** | 2 | **hijikake[hidʒikake]** | 4 | 7 |

IPA = International Phonetic Alphabet, PED = Phonological Edit Distance.

|  | Type | **Japanese Label [IPA]** | Mora | **English Label 1 [IPA]** | Syllables | **English Label 2 [IPA]** | Syllables | PED |
| --- | --- | --- | --- | --- | --- | --- | --- | --- |
| 1 | DE | **kuchibashi[kutʃibaʃi]** | 4 | **beak[bik]** | 1 | **bill[bɪl ]** | 1 | 2 |
| 2 | DE | **mame[mame]** | 2 | **beans[binz]** | 1 | **peas[piz]** | 1 | 2 |
| 3 | DE | **mimi[mimi]** | 2 | **crust[krʌst]** | 1 | **ear[ɪər]** | 1 | 5 |
| 4 | DE | **awa[awa]** | 2 | **bubbles[bʌbəlz]** | 2 | **foam[foʊm ]** | 1 | 6 |
| 5 | DE | **isu[isu]** | 2 | **chair[ʧeər]** | 1 | **stool[stul ]** | 1 | 4 |
| 6 | DE | **hasami[hasami]** | 3 | **claw[klɔ]** | 1 | **scissors[sɪzərz]** | 2 | 6 |
| 7 | DE | **kiba[kiba]** | 2 | **fang[fæŋ]** | 1 | **tusk[tʌsk]** | 1 | 4 |
| 8 | DE | **hari[haɽi]** | 2 | **hand[hænd]** | 1 | **needle[nidəl]** | 1 | 5 |
| 9 | DE | **tsuno[tsuno]** | 2 | **horns[hɔrnz]** | 1 | **antlers[æntlərz]** | 2 | 6 |
| 10 | DE | **nezumi[nezumi]** | 3 | **mouse[maʊs]** | 1 | **rat[ræt]** | 1 | 4 |
| 11 | DE | **hige[hige]** | 2 | **mustache[mʌstæʃ]** | 2 | **beard[bɪərd]** | 1 | 6 |
| 12 | DE | **tsume[tsume]** | 2 | **nail[neɪl]** | 1 | **claw[klɔ]** | 1 | 4 |
| 13 | DE | **oyayubi[ojajubi]** | 4 | **thumb[θʌm]** | 1 | **toe[toʊ]** | 1 | 3 |
| 14 | DE | **hane[hana]** | 2 | **trunk[trʌŋk]** | 1 | **nose[noʊz]** | 1 | 5 |
| 15 | DE | **tokei[tokei]** | 3 | **watch[wɑʧ]** | 1 | **clock[klɑk]** | 1 | 3 |
| 16 | DE | **su[su]** | 1 | **web[web]** | 1 | **nest[nest]** | 1 | 3 |

aIPA = International Phonetic Alphabet, PED = Phonological Edit Distance.
