# Supplementary material for: One Label or Two? Linguistic Influences on the Similarity Judgment of Objects between English and Japanese Speakers
Source: Front Psychol. 2017 Sep 26;8:1637. doi: 10.3389/fpsyg.2017.01637 (PMC5623002; doi:10.3389/fpsyg.2017.01637)

1. fukuro – kaban (bag – bag)


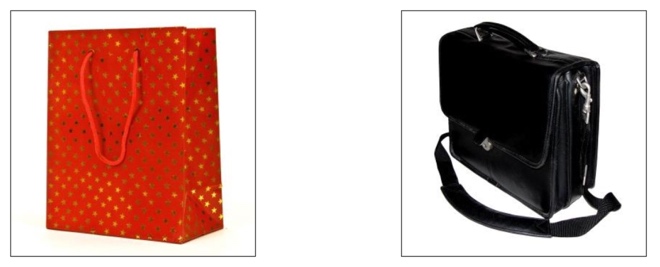


1. fukuro – kaban (bag – bag)


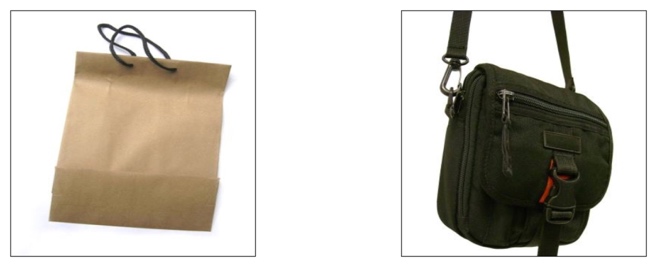


2. gen – ito (string – string)


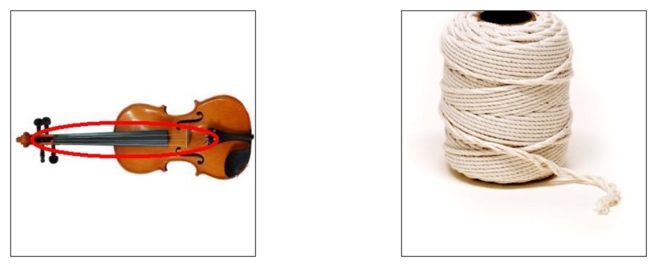


2. gen – ito (string – string)


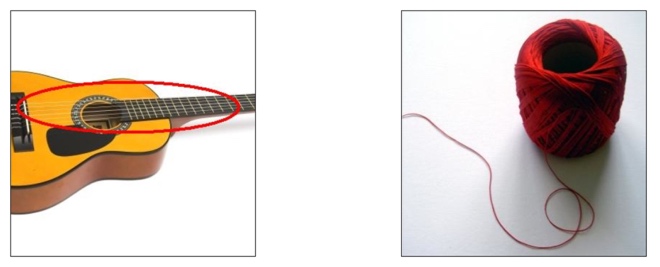


3. geto – mon (gate – gate)


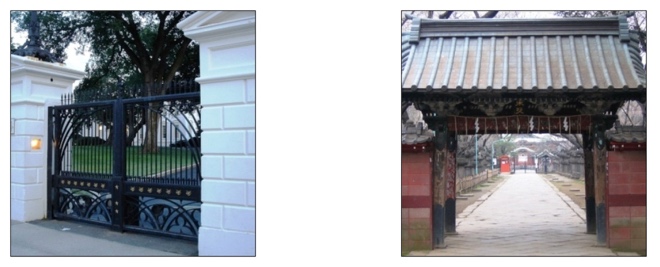


3. geto – mon (gate – gate)


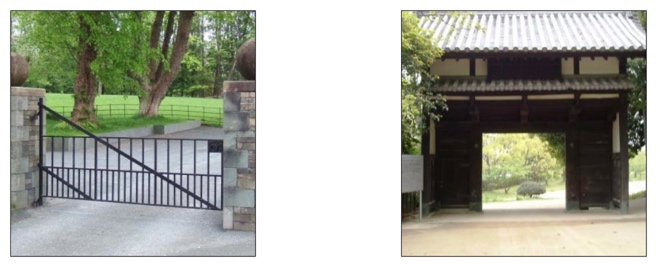


4. gunte – tebukuro (gloves - gloves)


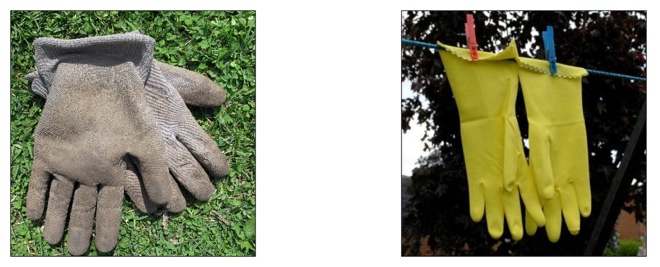


4. gunte – tebukuro (gloves - gloves)


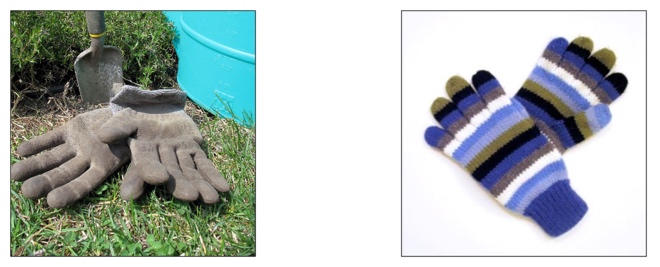


5. hake – fude (brush – brush)


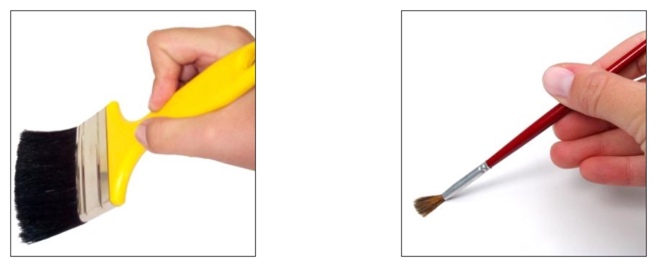


5. hake – fude (brush – brush)


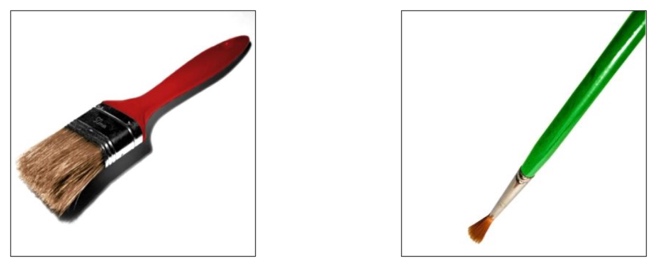


6. hei – saku ( fence – fence)


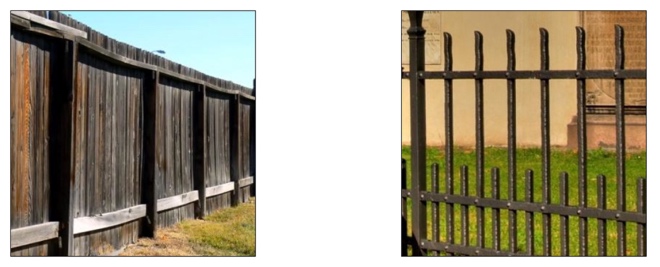


6. hei – saku ( fence – fence)


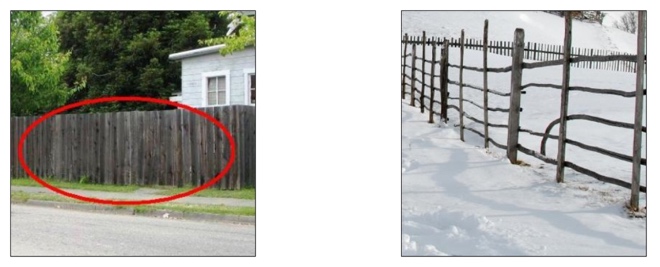


7. jaguchi – totte (handle – handle)


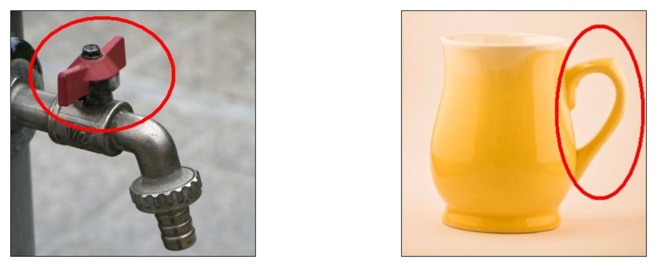


7. jaguchi – totte (handle – handle)


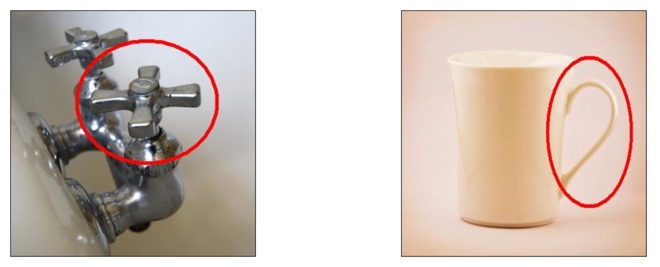


8. kankisen – sempuki (fan – fan)


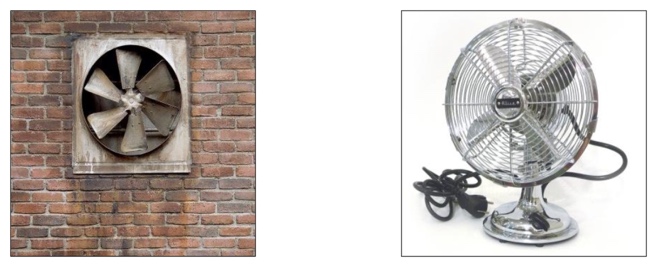


8. kankisen – sempuki (fan – fan)


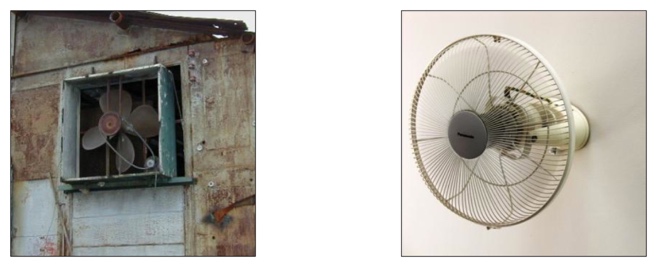


9. kikyu – fusen (balloon – balloon)


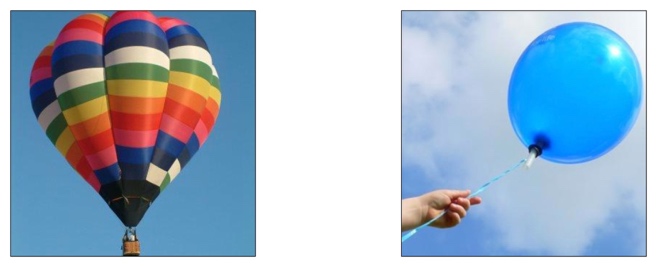


9. kikyu – fusen (balloon – balloon)


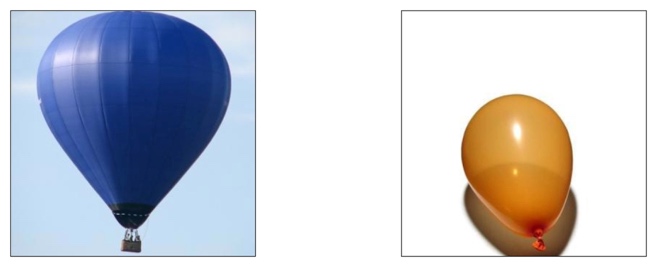


10. kitte – hanko (stamp – stamp)


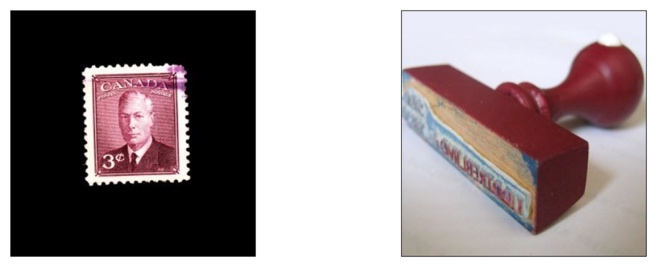


10. kitte–hanko (postage stamp–stamp)


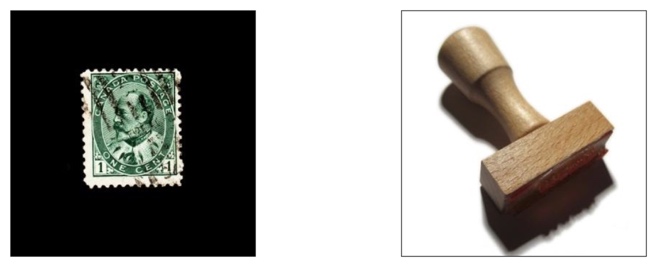


11. mizu – oyu (water – water)


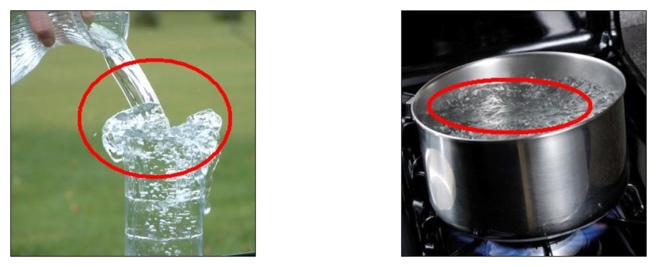


11. mizu – oyu (water – water)


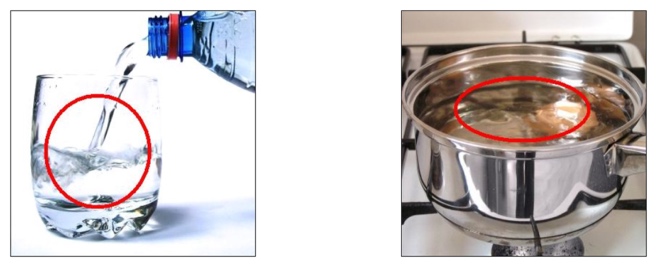


12. naifu – hocho (knife – knife)


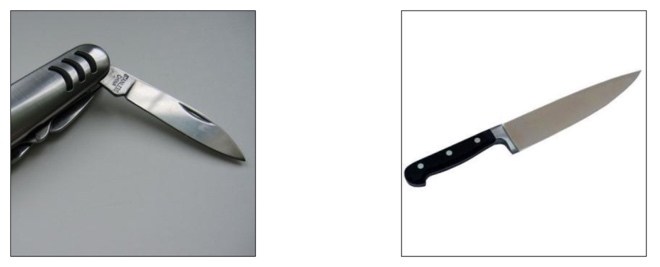


12. naifu – hocho (knife – knife)


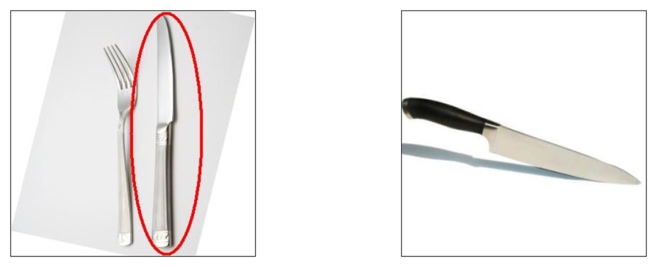


13. shokkaku – antena (antenna – antenna)


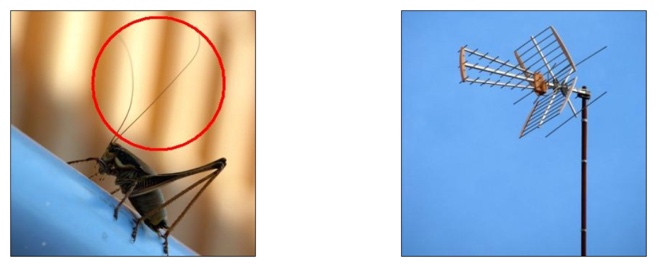


13. shokkaku – antena (antenna – antenna)


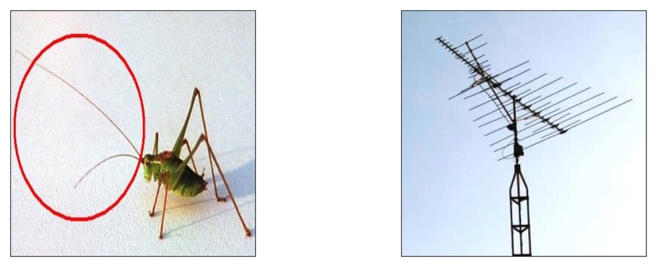


14. kara – kora (shell – shell)


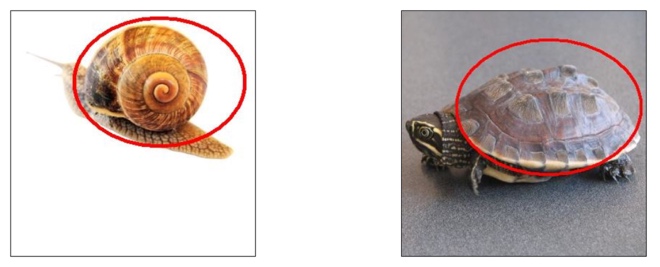


14. kara – kora (shell – shell)


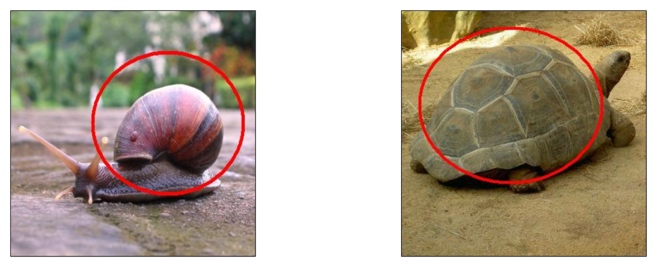


15. suiheisen – chiheisen (horizon – horizon)


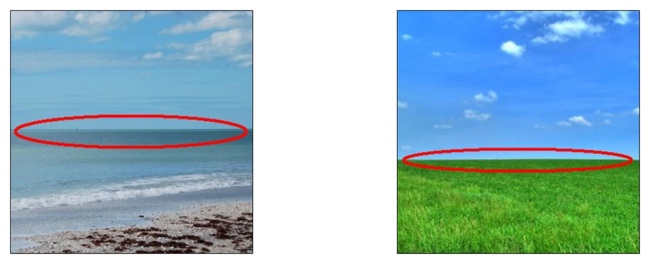


15. suiheisen – chiheisen (horizon – horizon)


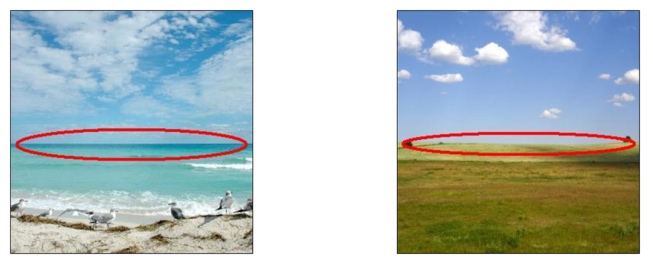


16. suzu – kane (bell – bell)


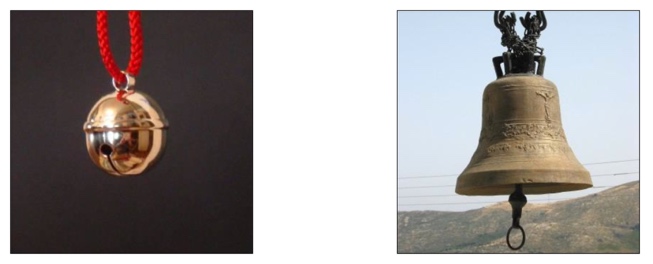


16. suzu – kane (bell – bell)


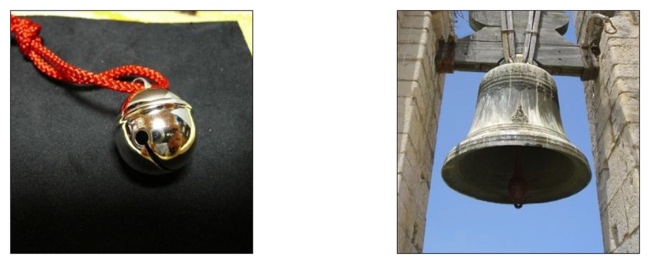


17. tsubasa – hane (wing – wing)


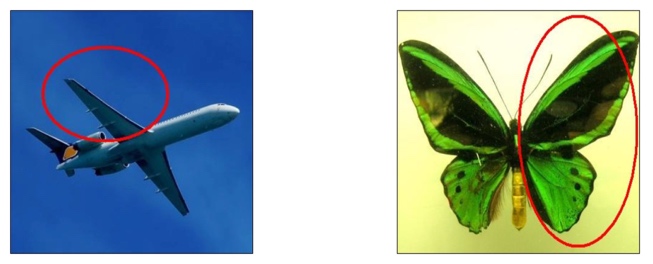


17. tsubasa – hane (wing – wing)


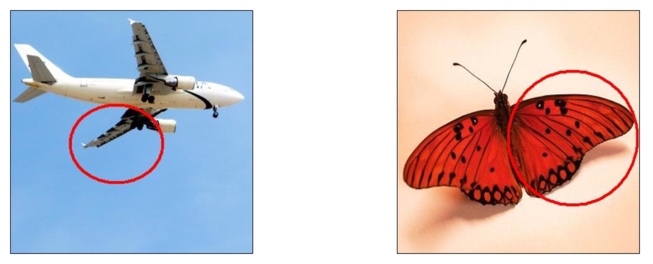


18. ude – hijikake (arm – arm)


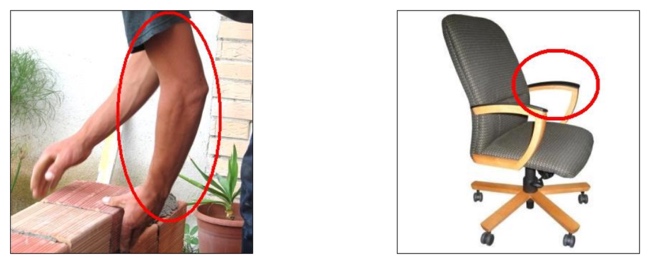


18. ude – hijikake (arm – arm)


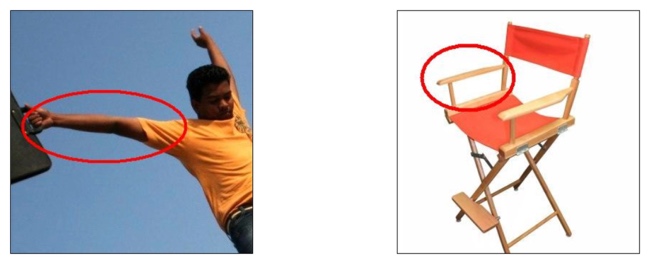


1. beak – bill (kuchibashi – kuchibashi)


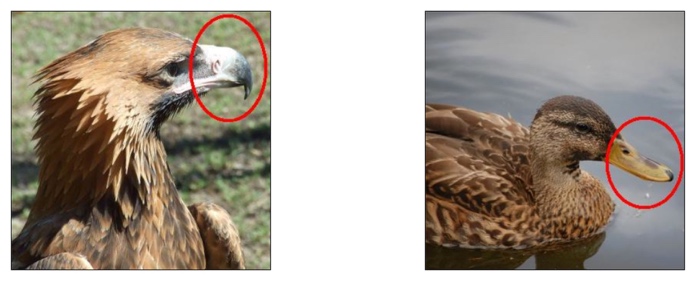


1. beak – bill (kuchibashi – kuchibashi)


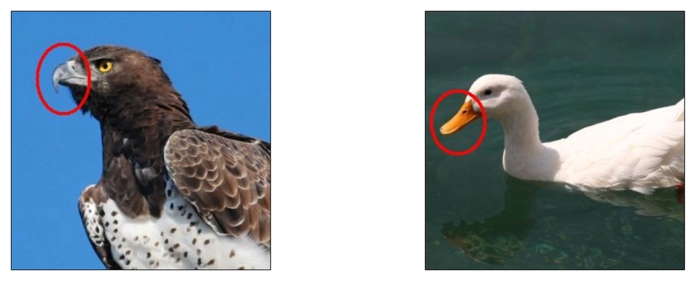


2. beans – peas (mame – mame)


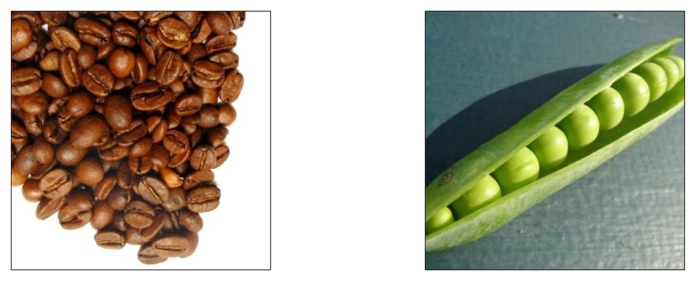


2. beans – peas (mame – mame)


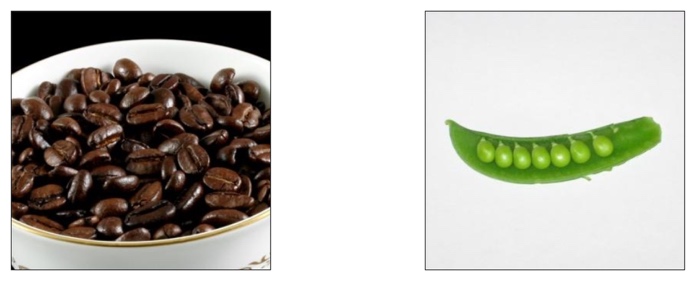


3. breadcrust – ear (mimi – mimi)


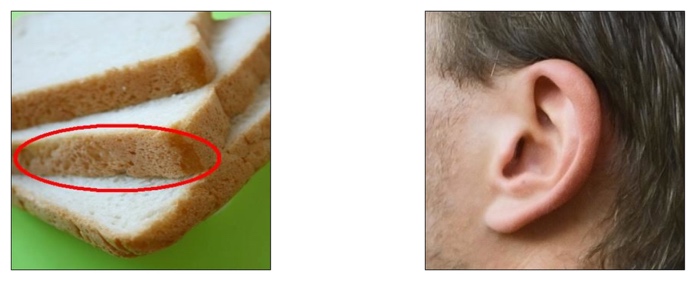


3. breadcrust – ear (mimi – mimi)


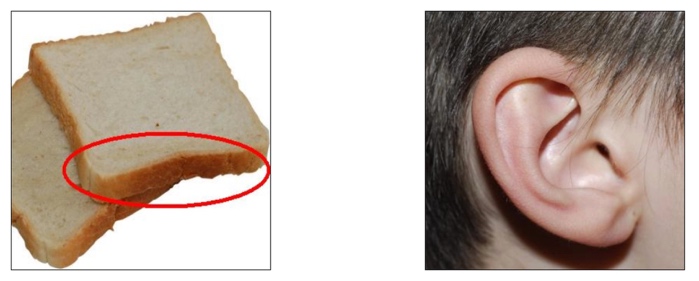


4. bubbles – foam (awa – awa)


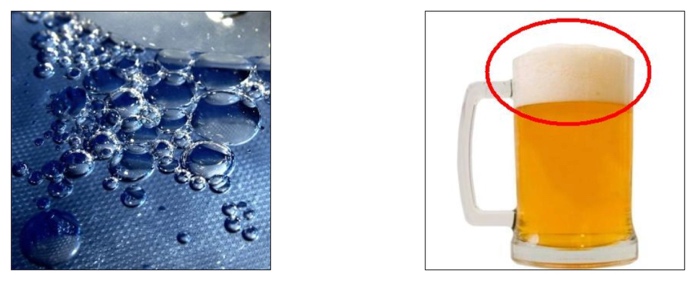


4. bubbles – foam (awa – awa)


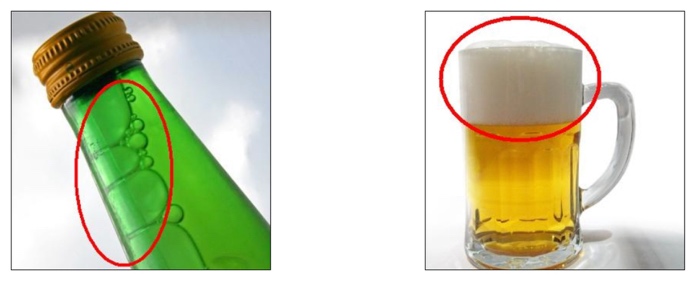


5. chair – stool (isu – isu)


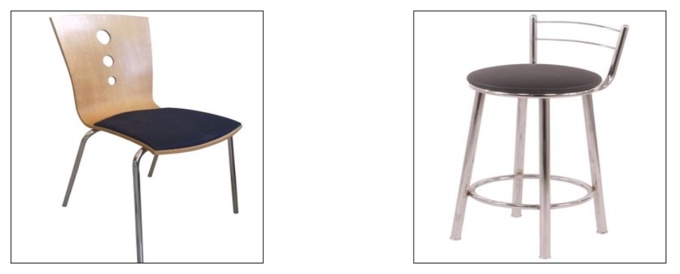


5. chair – stool (isu – isu)


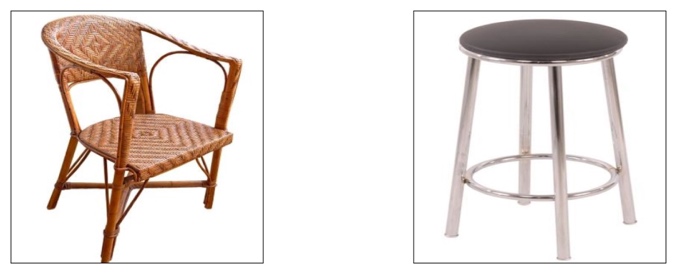


6. claw – scissors (hasami – hasami)


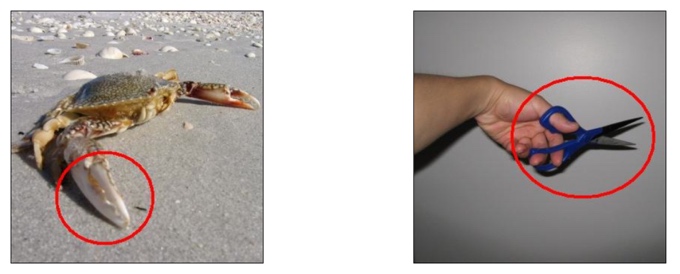


6. claw – scissors (hasami – hasami)


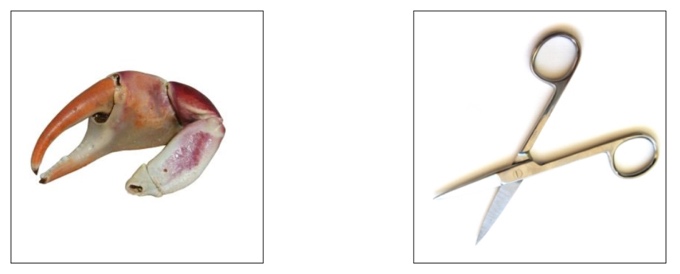


7. fang – tusk (kiba – kiba)


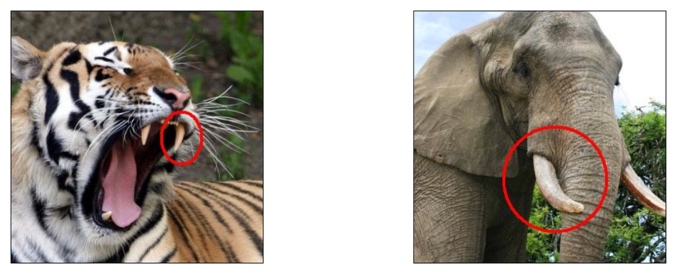


7. fang – tusk (kiba – kiba)


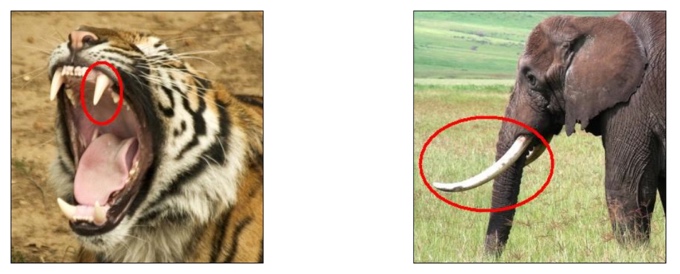


8. hand – needle (hari – hari)


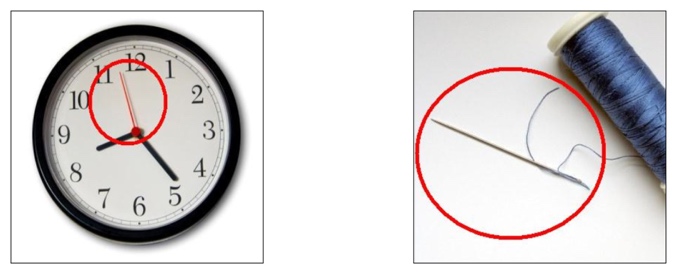


8. hand – needle (hari – hari)


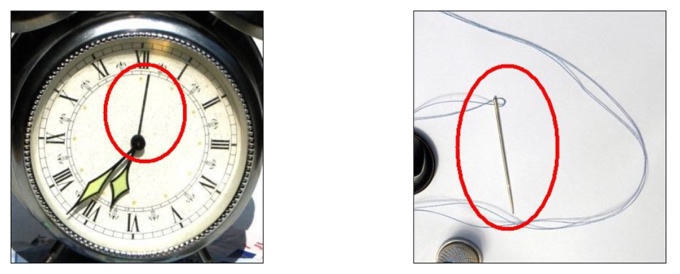


9. horns – antlers (tsuno – tsuno)


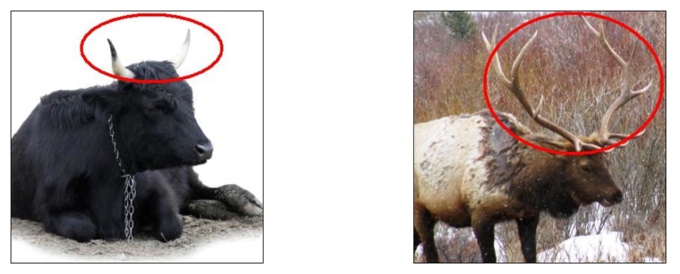


9. horns – antlers (tsuno – tsuno)


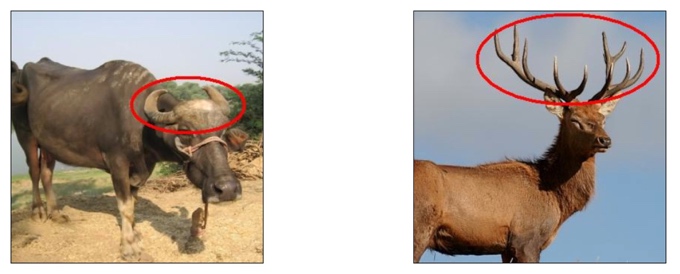


10. mouse – rat (nezumi – nezumi)


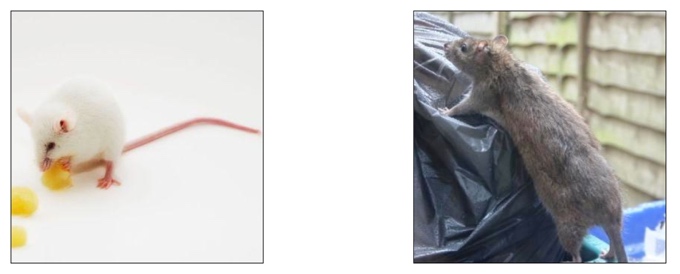


10. mouse – rat (nezumi – nezumi)


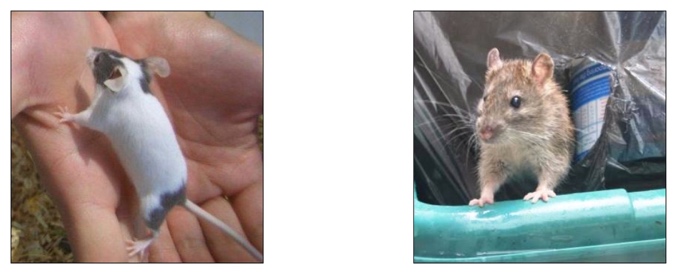


11. mustache – beard (hige – hige)


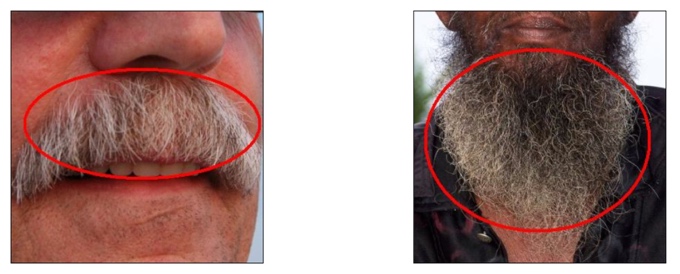


11. mustache – beard (hige – hige)


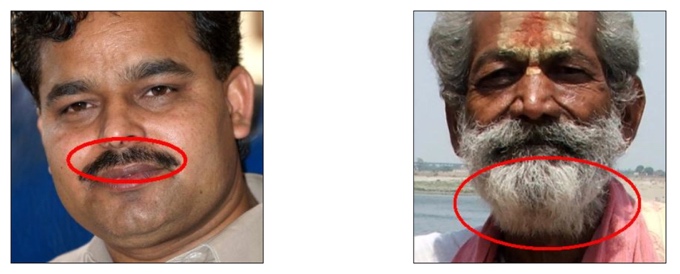


12. nail – claw (tsume – tsume)


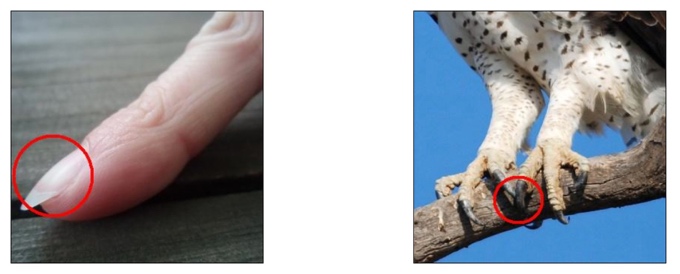


12. nail – claw (tsume – tsume)


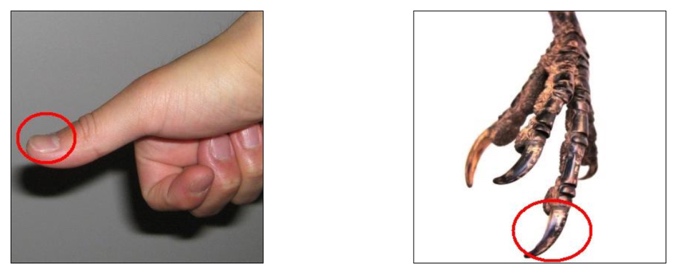


13. thumb – toe (oyayubi – oyayubi)


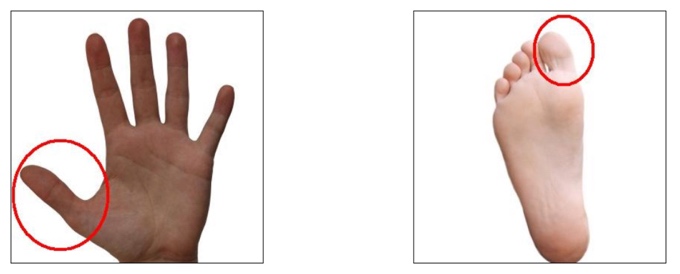


13. thumb – toe (oyayubi – oyayubi)


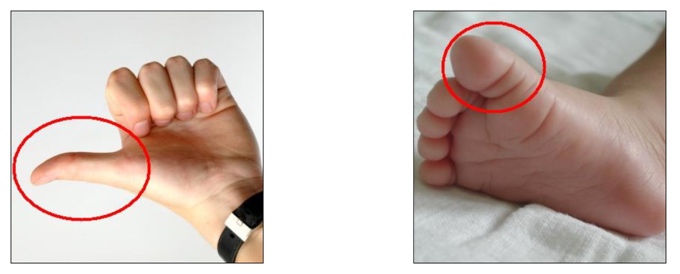


14. trunk – nose (hana – hana)


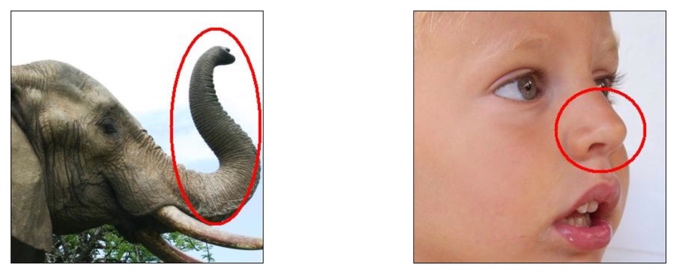


14. trunk – nose (hana – hana)


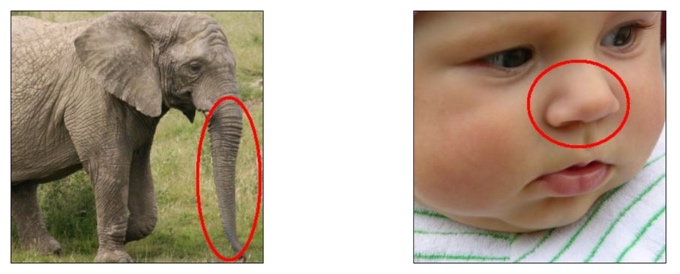


15. watch – clock (tokei – tokei)


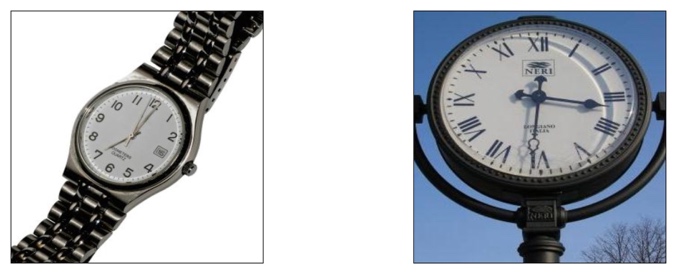


15. watch – clock (tokei – tokei)


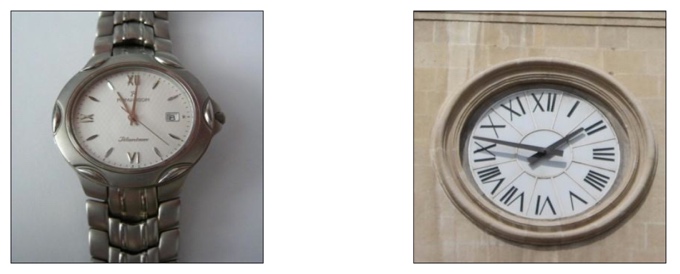


16. web – nest (su – su)


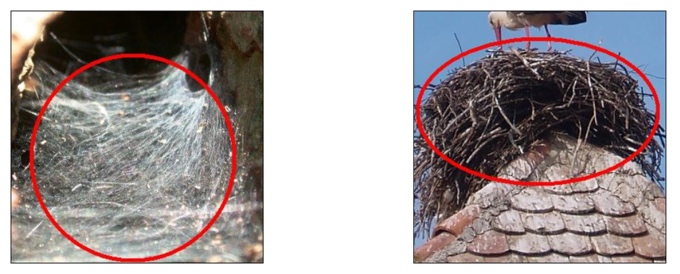


16. web – nest (su – su)


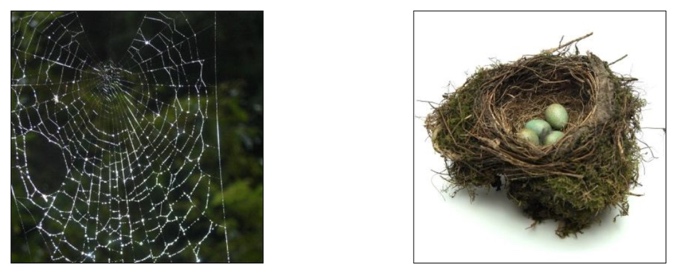


Identical filler 1


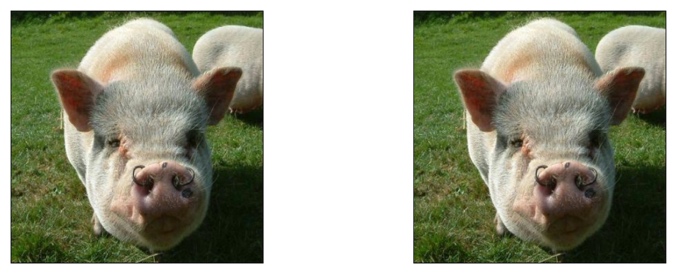


Identical filler 2


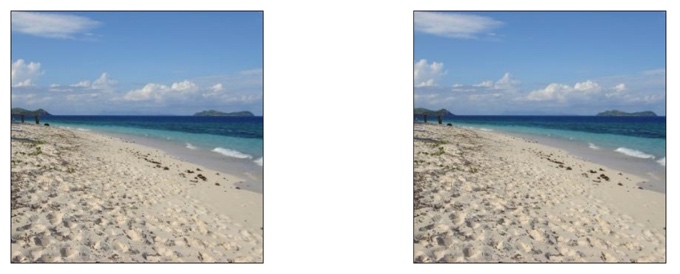


Identical filler 3


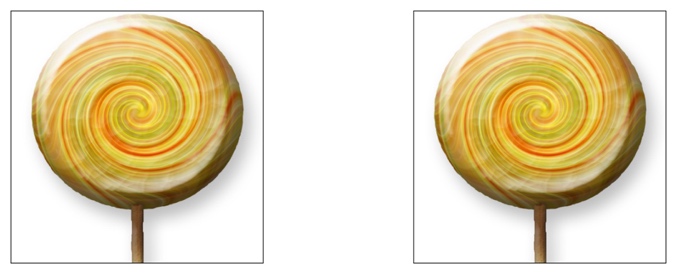


Identical filler 4


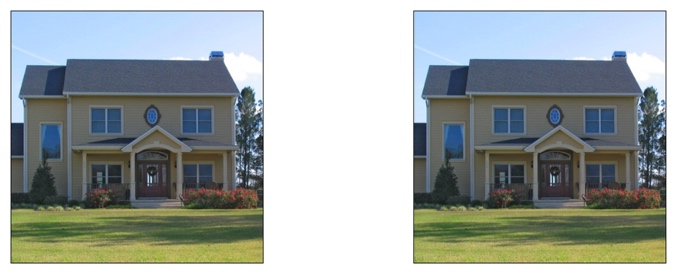


Identical filler 5


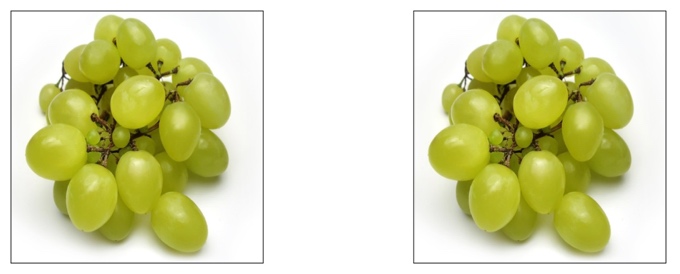


Identical filler 6


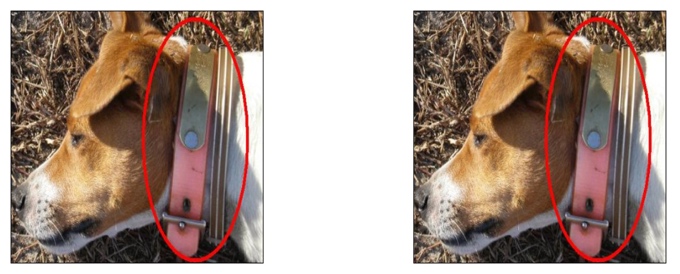

Supplement: Supplementary file 2 [file Data_Sheet_1.docx]
